# Shark genome size evolution and its relationship with cellular, life-history, ecological, and diversity traits

**DOI:** 10.1038/s41598-024-59202-4

**Published:** 2024-04-17

**Authors:** Mario Torralba Sáez, Michael Hofreiter, Nicolas Straube

**Affiliations:** 1 Ichthyology Section, Bavarian State Collection of Zoology (SNSB-ZSM), 81247 Munich, Germany; 2 Systematic Zoology, Department Biology II, Faculty of Biology, Ludwig Maximilian University of Munich (LMU), 82152 Munich, Germany; 3 Evolutionary Adaptive Genomics, Institute for Biochemistry and Biology, University of Potsdam, 14476 Potsdam, Germany; 4 Department of Natural History, University Museum Bergen, University of Bergen (UiB), 5007 Bergen, Norway

**Keywords:** Ecology, Evolution, Genetics, Zoology, Evolutionary ecology

## Abstract

Among vertebrates, sharks exhibit both large and heterogeneous genome sizes ranging from 2.86 to 17.05 pg. Aiming for a better understanding of the patterns and causalities of shark genome size evolution, we applied phylogenetic comparative methods to published genome-size estimates for 71 species representing the main phylogenetic lineages, life-histories and ecological traits. The sixfold range of genome size variation was strongly traceable throughout the phylogeny, with a major expansion preceding shark diversification during the late Paleozoic and an ancestral state (6.33 pg) close to the present-day average (6.72 pg). Subsequent deviations from this average occurred at higher rates in squalomorph than in galeomorph sharks and were unconnected to evolutionary changes in the karyotype architecture, which were dominated by descending disploidy events. Genome size was positively correlated with cell and nucleus sizes and negatively with metabolic rate. The metabolic constraints on increasing genome size also manifested at higher phenotypic scales, with large genomes associated with slow lifestyles and purely marine waters. Moreover, large genome sizes were also linked to non-placental reproductive modes, which may entail metabolically less demanding embryological developments. Contrary to ray-finned fishes, large genome size was associated neither with the taxonomic diversity of affected clades nor with low genetic diversity.

## Introduction

Sharks (Chondrichthyes: Selachii) are widely known for their ecological importance as apex as well as mesopredators in aquatic ecosystems^1^. Believed to have originated in the late Permian^2^, the diversity of modern sharks comprises more than 500 described species grouped in two superorders: Galeomorphii (with four orders) and Squalomorphii (with five)^3^. Despite their high evolutionary adaptability, reflected by their circumglobal distribution further spanning a plethora of habitats and life-history traits, few genomic resources are available for this clade^4,5^.

One obstacle to the sequencing and annotation of many shark genomes lies in their overall large size. Among vertebrates, shark genomes are exceeded in size only by those of lungfishes (Dipnoi) and salamanders (Urodela)^6^. There are at least 15 known species of sharks with “C-values” (haploid DNA content in picograms) > 10 pg, where 1 pg = 978 Mbp^6^. Most of these large genomes are present within the Squalomorphii clade (4–17 pg)^7^. In contrast, galeomorph sharks are characterized by DNA contents varying within a distribution range (2.5–7.5 pg) similar to that of rays, skates and sawfishes (Chondrichthyes: Batoidea)^7^, the sister group to the Selachii, which together form the subclass Elasmobranchii^8^. Its sister clade, the Holocephali (or chimaeras)^8^, is characterized by the lowest genomic contents within Chondrichthyes (1.5–1.9 pg)^7,9^. Despite their variability in bulk DNA, the evolutionary forces that have shaped shark genome size diversity remain poorly studied^5,10^.

To date, several molecular mechanisms by which genomes can expand and/or shrink have been uncovered^10^. In some cases, C-values change in a more or less punctuated fashion, a pattern intuitively associated with events of polyploidy^11^. A previous study revealed that both batoid and galeomorph genome sizes tend to vary around two modal values (3.5 and 7 pg), following a doubling series, and that several squalomorph species have C-values twice the lowest genome sizes in the group^12^. Multiples of 1.45 pg in chondrichthyan genome size variation have also been reported in more recent studies^9^. Although these patterns may have been a sampling artefact, comparisons of chondrichthyan karyotypes have hinted at the general significance of polyploidy early in the diversification of this group^7,13^. However, recent genome-wide analyses have rejected this idea^14,15^, making the further study of shark karyotype evolution of such discontinuous patterns well warranted.

Regardless of the mechanisms of change, genome size has been found to be positively correlated with cell and nucleus sizes and inversely correlated with cell division rate in a causative manner across distantly related taxa^10,16^. These universal cytogenomic relationships result, by extension, in strong correlations with organism-level traits of selective importance, suggesting adaptive consequences of the bulk properties of the genome independent of its respective nucleotide sequence (the *nucleotype* hypothesis)^16^. For example, changes in cell size will have an immediate effect on body size in organisms with a constrained number of cells. As such, body size has been found to scale with genome size in some invertebrates, birds, rodents, and bats^17^. Cell size variation can also exert an effect on aerobic metabolism, for as size increases, relative surface area and hence, gas exchange efficiency, decreases in erythrocyte cells^18^. In fact, a negative association between genome size and basal metabolic rate has been found in birds^19,20^ and mammals^21^. Notably, some of the smallest vertebrate genomes exist within volant taxa, for which high metabolic rates are prerequisites for powered-flight^10,22^. Additionally, the (negative) influence of DNA content on cell division rate (as larger genomes demand more time for gene expression and replication^16^) should translate into negative associations of genome size with the rate or complexity of development of an organism, a pattern reported for some insects, crustaceans, and amphibians^10^.

The existence of ecological patterns of genome size variation further emphasizes its adaptive significance, since it is indicative of natural selection acting on the organismal phenotypes indirectly modulated by DNA content via its effects on cellular parameters. A positive association between C-value and thermal regime has been noticed in several taxonomic groups (e.g., certain plants, zooplankton, crustaceans, salamanders, etc.), with those species living at high latitudes or altitudes exhibiting greater C-values as well as more frequent cases of polyploidy than their low latitude/altitude counterparts^6,10,11,23^. This trend is consistent with the different reproduction strategies (r- and K-strategies) commonly associated with different thermal regimes^18^. The rapid metabolism and high developmental rates of short-lived r-selected organisms (which can be better achieved if cells and genomes are small) are features selected under the ephemeral conditions imposed by warmer climates. On the contrary, cold environments favour K-selected organisms^24^, characterized by a set of traits (low metabolic rate, slow development, late maturity, long lifespan, and low fecundity) not selected against or potentially favoured by larger genomes. Hardie and Hebert^25^ observed this pattern in both cartilaginous (accounting for 16 shark species) and ray-finned fishes (Actinopterygii), in which cell and genome sizes were larger in polar and/or bathypelagic (i.e., cold) environments, consistent with the observed increase in genome size with water-depth in argentinoid fishes^26^. However, later studies including denser taxonomic sampling of actinopterygian fishes found either the contrary pattern^9^ or the same result only as an artefact of polyploidy or driven by phylogenetic proximity^27^, raising doubts about the influence of temperature on fish genome size modulation. Along the same lines, it has been argued that organisms with broader ecological tolerance tend to show larger genome sizes (potentially as a buffer against fluctuating conditions^23^) than more specialized forms living in stable environments^28^.

Interestingly, despite the apparent adaptive implications of DNA content, the negative trend between the number of species in a clade and its average genome size reported for actinopterygian fishes^27^ and vertebrates^29^ suggests that large genomes may adversely affect species diversification (evolvability) in the long term. Another theory postulates that genome size is rather a result of genetic drift (the *mutational-hazard* hypothesis)^30^. In this regard, in taxa with sustained small populations, slightly-deleterious duplicated genes and transposons will more readily become fixed by drift and consequently, these species will come to harbour larger genomes. This hypothesis thus predicts an inverse relationship between genome size and population size.

Overall, most previous comparative analyses on the evolution of fish genome sizes have been either entirely focused on ray-finned species or strongly biased toward them, showing contrasting results across ecological gradients. Therefore, the suggestion that the larger genomes found in chondrichthyan relative to actinopterygian fishes^9,25^, or within sharks in Squalomorphii relative to Galeomorphii^13^ (or rather, in deep-sea relative to shallow water inhabitants^5,13^) are associated with their respective more K-selected lifestyles requires further investigation. We used published genome size estimates for 71 shark species representing the main phylogenetic lineages, life-histories, and ecological niches. In order to provide a better understanding of the proximate causes and ultimate consequences of shark genome size evolution, we performed phylogenetic comparative analysis involving the reconstruction of ancestral states for both genome size and chromosome number estimates, together with the characterization of their mode and rate of evolution and the assessment of potential relationships between genome size and a wide array of karyotype, cell, life-history, ecology and diversity-related parameters.

## Methods

### Data compilation

Standardized genome size C-values (haploid DNA content per cell measured in picograms, pg/n) were collected from the Animal Genome Size Database (Release 2.0)^31^, the Squalomix consortium data repository^14,32,33^, and the literature^7,13,34^ (and references therein), excluding values from potential triploid cytotypes or hypertrophic cells. Measurement technique was shown not to significantly influence species-specific genome size estimations across vertebrates (mixed-effects ANOVA [fixed effect: measurement technique; random effect: species]: *F* = 0.65, *p* = 0.597, *n*_estimates_ = 20, *n*_groups_ = 5; R package *nlme* v. 3.1.164^35^; data from the Animal Genome Size Database). Therefore, multiple records were averaged in order to provide a single C-value per species (only needed for 18 species; Supplementary Table S1). Genome size estimates derived from published whole-genome sequencing outputs (like whole genome assembly length or estimates from k-mers) were disregarded, given their scarce availability together with (1) their tendency to underestimate the size of the genomes they represent^33,36^, and (2) the availability of alternative genome size estimates for the same species measured with more reliable, non-sequencing-based techniques (see Supplementary Table S11). Karyotype information, based on somatic cells, was obtained from the literature^7,13,15,34,37–40^ (and references therein) and included: haploid chromosome numbers (n), fundamental chromosome arm numbers (FN), and the numbers of two-armed (atelocentric) and one-armed (telocentric) chromosomes, from which the ratio was used as a metric for karyotype composition (more information in Supplementary Table S1). Updates to the species names and higher taxonomy from older references were applied following the bibliographic database Shark-References^3^.

Genome size data were compared against a wide variety of biological parameters related to the karyotype (aforementioned), cytology, life-history, ecology, and taxonomic and genetic diversity of sharks. Biological data were mostly extracted from the literature^8,41–47^ (and references therein) and the online databases Cell Size Database^48^, AnAge^49^, FishBase^50^, Shark-References^3^, and IUCN Red List^51^.

Cell and nucleus sizes were represented by erythrocyte dry smear estimates of cell area (*C*_*a*_) and nucleus area (*N*_*a*_), respectively. Body size was assessed by (1) total body length (*L*) and (2) maximum body weight (*W*_*max*_). For *L*, a Principal Component Analysis (PCA, implemented in R) was performed on the estimates of minimum and maximum common body length at maturity for males and females and of maximum recorded body length for each species. The resulting coordinates of the first principal component (i.e., PCA1 scores), which summarized 96.36% of the length data variation, were used as representatives for total body length (*L*_*PCA1*_). Body form was represented by (1) precaudal body shape (*PBS*, body types 1 to 4 following Thomson and Simanek^52^, with an additional body type 5 assigned to dorsoventrally flattened species), and (2) caudal fin aspect ratio (*CFAR,* caudal fin height squared divided by its surface area). Physiological factors included (1) standard metabolic rate (*SMR*), measured as mass-specific oxygen consumption rate at rest during fasting (corrected to 20 °C using a fish-specific temperature coefficient, Q_10_ = 1.65^53^) as well as (2) body-size corrected tail shape (*TS*, tail types 1 to 4 following Thomson and Simanek^52^) as a proxy for cruising speed^54^. Developmental and demographic parameters included (1) the growth completion rate (*k*) from the von Bertalanffy growth model; (2) age, for which we used the PCA1 scores (*T*_*PCA1*_) of a PCA performed on the species-specific estimates of age at maturity for males and females and of maximum recorded lifespan, summarizing 98.36% of the age data variation; and (3) maximum intrinsic rate of population increase (*r*_*max*_, a standard measurement of population productivity). As for reproductive factors, we included reproduction mode (coded as “oviparous”, “aplacental viviparous”, and “placental viviparous”), and average litter size (*L*_*s*_). Regarding species ecology, we accounted for preferred water temperature and depth (including average depth and maximum depth range), climate (“tropical”, “subtropical”, “temperate”, “boreal”, and “worldwide”), occurrence across the water column (“pelagic”, “benthopelagic”, and “epibenthic”), habitat (“reef-associated”, “coastal”, “oceanic”, and “deep-water”) and salinity (“marine”, “marine-brackish”, and “amphidromous”). Regarding diversity parameters, taxonomic diversity was assessed by the number of species per family and per order, the number of genera per family and per order, and the number of families per order. Finally, the level of neutral genetic diversity was represented by expected heterozygosity (*H*_*e*_) and used as a proxy for effective population size (*N*_*e*_). For more information regarding variable definitions, detailed references used, and visualizations of the data distribution (for quantitative variables) and category assignment (for categorical ones), see Supplementary Methods and Figs. S1 and S2.

### The phylogeny

In order to apply phylogenetic comparative methods, we generated a phylogeny for all shark species included in our main dataset, with four chimaera and one batoid species designated as the outgroup for ancestral reconstruction analyses (species listed in Supplementary Table S1), from the most comprehensive time-calibrated molecular phylogeny of all extant Chondrichthyes available to date^55^. More specifically, we downloaded 100 trees (pruned to the species of our study) randomly sampled from the 10,000 alternative trees available on http://www.sharktree.org under the “Full resolved 10 fossil” pseudo-posterior distribution. We then summarized the sample of 100 trees onto a single ‘Maximum Clade Credibility’ (MCC) tree using TreeAnnotator within the BEAST v. 2.7.6 software package^56^. We used the MCC tree as the backbone phylogenetic hypothesis for subsequent analyses (“Statistical analysis” section), while repeating and summarizing the analyses across the 100 alternative trees to assess the robustness of results to variation in tree topology and branch lengths (except for “Chromosome number evolution” section, due to computational limitations). All the remaining analyses were performed in R v. 4.3.2^57^. Tree handling involved the R packages *ape* v. 5.7.1^58^ and *phytools* v. 2.1.1^59^.

### Statistical analysis

#### Mode and rate of genome size evolution

To reconstruct ancestral states of genome size, first a total of three maximum likelihood (ML) univariate models of trait evolution were fitted to the (ln-transformed) genome size data, whether including or excluding the outgroup species, using the *geiger* R package v. 2.0.11^60^. These included (1) the ‘Brownian Motion’ (BM) model, which describes the evolution of a continuous quantitative trait as a random walk^61^; (2) the ‘Ornstein–Uhlenbeck’ (OU) model, commonly used to model stabilizing selection by creating a tendency toward a range of phenotypic values, thus indicating an adaptive optimum^62^; and (3) the ‘Early-Burst’ (EB) model, where the greatest phenotypic divergence occurs early in the tree^63^. This step required the random resolution of soft polytomies (via the 'multi2di' function of the same package). Simulation-based studies have highlighted the importance of incorporating an estimate of error in trait measurements to avoid bias toward models with lower phylogenetic signal^64^ (e.g., OU model; see definition for phylogenetic signal below). As such, genome size standard errors (SE) for species with more than one available estimate (after ln-transformation) were incorporated into the analysis, following Pennell et al.^60^, and parameter estimates optimized after 1,000 random restarts. Model selection was based on the Akaike Information Criterion weights (AICw), which show, among a set of competing models, the conditional probability of each model at providing the best explanation^65^. The (ln-transformed) ancestral genome size estimates were then calculated under the best-fitting model parameters via ML, with 10,000 iterations for optimization, and the (untransformed) values colour-coded onto the branches of the MCC tree [*phytools* package].

In addition, Pagel’s lambda (λ), delta (δ), and kappa (κ) tree transformation parameters^66^ were calculated via ML to further test for potential deviations from a constant-rate process of evolution (i.e., BM model) [*geiger* package]. Specifically, Pagel’s λ measures the strength of phylogenetic signal, meaning the degree to which variation among species trait data is dependent on their phylogenetic proximity (with λ = 1 when the phylogeny fully underlies trait variability and λ = 0 for traits varying independently of their evolutionary history). Pagel’s δ allows for determining whether the rate of evolution slowed down (δ < 1), remained constant (δ = 1), or accelerated (δ > 1) through time. Pagel’s κ captures patterns of punctuated evolution along the tree (with character change being concentrated at speciation events for κ = 0—*punctuated evolution*—or evolving *gradually* when κ = 1). We followed the same steps for incorporating measurement error and parameter optimization as aforementioned. *P*-values were calculated via likelihood-ratio test (implemented in R), with the BM model (where λ = 1, δ = 1, and κ = 1), but also models where λ = 0 and κ = 0 (using the ‘rescale’ function of the same package) as null hypotheses and assuming a *chi*-squared distribution with one degree of freedom (*df*, equal to the difference in the number of parameters between the models compared). Estimations of all the Pagel’s parameters were applied to data including and excluding the outgroup species.

Finally, the rates at which genome size evolved in galeomorph and squalomorph sharks were compared. For that, Phylogenetically Independent Contrasts of Felsenstein^67^ (PICs) were calculated for each clade [*ape* package] and their absolute values compared using a two-sided Mann–Whitney U test (implemented in R; note that PICs are based on a BM model of evolution, which was the best-fitting model of genome size evolution when only sharks were included in the analysis; Supplementary Table S2). Given that the magnitude of independent contrasts compiles information regarding the degree of character change along the branches of the tree, subsets of the tree with large (absolute) PICs are indicative of concentrated, high rates of evolution^67^. For this step, estimates were not ln-transformed, as this would remove any proportional dependency between genome size and the rate at which it evolves^68^.

#### Chromosome number evolution

Ancestral haploid chromosome numbers (n) were reconstructed, based on a continuous-time Markov process, using ChromEvol v. 2.0^69,70^. All models that did not require root chromosome number specification (Supplementary Table S4) were fitted to the karyotype data under the MCC phylogenetic hypothesis (species with unknown karyotype states—but known genome size—were coded with “X”, indicating missing data). Each model is characterized by a different set of parameters that relate to four possible mechanisms by which chromosome numbers can change, including: events of dysploidization, which can be ascending (via single chromosome gains) or descending (through chromosome losses), duplications (i.e., polyploidizations), and demi-duplications. These models allow for polytomies and polymorphic character states. As such, when several alternative chromosome counts existed for a single species (averaged for summary statistics and comparative analysis; see below), the relative frequency of each report was used to obtain the probability of each character state, which was then integrated into the model. For a reliable ancestral reconstruction, the total MCC tree length was adjusted to 1 before running ChromEvol, as previous simulation-based studies have shown that larger root-to-tip distances can lead to overestimation in the inferred number of transition events^69^. For each model, after the optimum rate parameter values were estimated during the first run (ML approach), a total of 10,000 simulations were used to compute the probability of any given chromosome number to exist at any internal node, together with the expected frequency of each chromosome number transition type along each branch (Bayesian approach). The range of chromosome values allowed in the simulations was set to 1 unit lower and 10 units larger than the range of empirical values observed in the dataset (i.e., 28–64). Model selection was based on the Akaike Information Criterion (AIC)^71^. ChromEvol results were plotted using the R packages *phytools* and *colorRamps* v. 2.3.1^72^.

#### Comparative analysis

##### Simple regression analysis

Summary statistics for genome size and karyotype parameters were calculated using standard methods. One-way ANOVA was used to test for differences in (ln-transformed) species average genome size among taxa, with significantly different groups identified through Tukey’s HSD (Honestly Significant Difference) test, using the R packages *car* v. 3.1.2^73^ and *multcomp* v. 1.4.25^74^, respectively. Relationships between genome size and the biological parameters described in “Data compilation” section, as well as the interrelationships between karyotype parameters, were assessed for sharks only. Prior to analysis, ln-transformations were applied where required to (1) *response* variables, in order to achieve normal or near-normal distributions (as assumed by the models implemented), tested via Shapiro–Wilk test and qq-plots (implemented in R) as well as to (2) quantitative *predictor* variables, in order to obtain linear relationships in *response vs. predictor* comparisons, assessed via scatter-plots. Given that shared ancestry can result in phylogenetic covariance of species data (and thus, the residuals of the models), phylogenetic corrections were incorporated when assessing all the relationships via Phylogenetic Generalized Least Squares (PGLS) regression analysis using the R package *caper* v. 1.0.3^75^. The PGLS approach allows for the estimation of Pagel’s λ from the least squares regression residuals via ML and the removal of the covariance due to phylogeny from the error structure of the model^76^. For each model, the likelihood surface of λ was visually inspected to avoid cases of local optima. All the comparative analyses of “Simple regression analysis” section were also tested via conventional (non-phylogenetically corrected) Ordinary Least Squares (OLS) regression analysis for comparison with previous reports on other taxa (for which more details and summary statistics are given in Supplementary Methods and Table S5, respectively).

To correct for body size where appropriate (i.e., *CFAR*, *SMR*, *TS*, *k*, *T*_*PCA1*_, *r*_*max*_, and *L*_*s*_; based on the literature^8,46,54,77,78^ and the correlation structure of our data; Supplementary Fig. S3), *L*_*PCA1*_ (1) was incorporated as a covariate in simple multivariate models (i.e., multiple regression, Analysis of Covariance or ANCOVA), or (2) was factored out via partial correlation in simple univariate models (for visual purposes, while ensuring that the same conclusions were reached when applying step 1). For *SMR*, this was done using the average mass of the individuals used in the original *SMR* estimation studies as a proxy for body size, given the big influence of body size on metabolism also through development^8^. Depth corrections (applied to climate, occurrence, and salinity) were applied following step 1 with (ln-transformed) average depth as the covariate (see further explanations in Supplementary Methods). Finally, we factored out the effects of *L*_*PCA1*_ and *T*_*PCA1*_ (following step 1) in an additional analysis to better disentangle the relationship between *H*_*e*_ and genome size. This is because *H*_*e*_, used here as a proxy for *N*_*e*_, is the product of *N*_*e*_ and the mutation rate (*µ*) at equilibrium, and *µ* can covary with body size and generation time^79^. Details for all the models analysed are available in Supplementary Table S5.

For categorical predictors, parameter effects were summarized using type II sum of squares (*F*-test). Post hoc analysis for identifying significant differences between pairs of group means is not currently available for PGLS analysis. As such, when the effect (*F*-test) of a categorical predictor rendered a significant *p*-value (i.e., < 0.05), post hoc pairwise comparisons between all of its categorical levels were manually applied using a re-ordering approach (i.e., re-running the PGLS model while setting a different category as the reference level—*intercept*—each time)^80^. We applied False Discovery Rates (FDR, implemented in R) to the resulting *p*-values to correct for the multiple comparisons performed during these post hoc pairwise comparisons.

##### Multivariate regression analysis

Finally, to prevent model overfitting, we used minimization of the AIC corrected for small sample sizes (AICc) to select the best-fitting minimum adequate multivariate models (those with Δ_i_ < 2, where Δ_i_ = AICc_i_ − AICc_min_)^71^ from all possible combinations of life-history and ecological variables in fully-combined models using the *MuMIn* R package v. 1.47.5^81^. Among all life-history and ecological parameters, the variables *W*_*max*_, *CFAR*, *SMR*, *k*, *T*_*PCA1*_, and *r*_*max*_ were excluded from the analysis to avoid sampling artefacts, given their scattered availability (Supplementary Fig. S1). However, these parameters are represented in the fully-combined models elsewhere. *L*_*PCA1*_ was set as a fixed covariate to control for allometry during all permutations. Prior to model selection, (multi-)collinearity among predictors was assessed via Generalized Variance Inflation Factor (GVIF^1/(2⋅df)^) analysis in fully-combined models [*car* package]. Categorical predictors with aliased coefficients (i.e., predictors with perfect collinearity between at least one of their categories, namely *PBS* and *TS,* occurrence and habitat; Supplementary Fig. S2) were analysed separately (models 1 to 4 in Table 3), while predictors with GVIF^1/(2⋅df)^ > 2 (i.e., preferred water temperature, average depth, and depth range) were not allowed to co-occur during model selection (see Supplementary Fig. S3 for a visualization of the correlation structure among quantitative predictors). Similarly, the predictors *PBS* and *TS* were not allowed to co-occur with occurrence or habitat, given that certain morphologies are commonly associated with specific environments. When the effect (*F*-test) of a categorical predictor had a *p*-value < 0.05 among the best multivariate models, post hoc analysis (as described above) was performed on the averaged set of best models obtained from the same fully-combined model where the given predictor appears (i.e., post hoc analysis on the conditional average of the best models’ parameter estimates). The set of predictors included among the best models with a *p*-value < 0.05 was the same as that obtained when applying a stepwise step-up model selection procedure. This guarantees the robustness of our model-fitting results and explains why no correction of *p*-values for the multiple comparisons involved in multivariate models was necessary^80^.

For all the comparative analyses described in “Comparative analysis” section, statistical significance was set at α = 0.05 (*) [very significant at α = 0.01 (**) and highly significant at α = 0.001 (***)]. Distribution of residuals (normality and homoscedasticity) was validated through visual inspection of model evaluation plots. Outliers, visually identified from *response vs. predictor* plots (before model fitting) or from cases with standardized residuals *|z| *> 3 (after model fitting)^82^, were eliminated from the analysis when found. Visualization of all the comparative analyses involved mainly the R packages *phytools*, *ggplot2* v. 3.5.0^83^ and *ggpubr* v. 0.6.0^84^.

All the data generated during this study, together with the R-script, are available in the figshare repository at https://figshare.com/s/e8fb3790edb95f605576.

## Results

### Genome size diversity in Chondrichthyes and sharks

**Figure 1:**
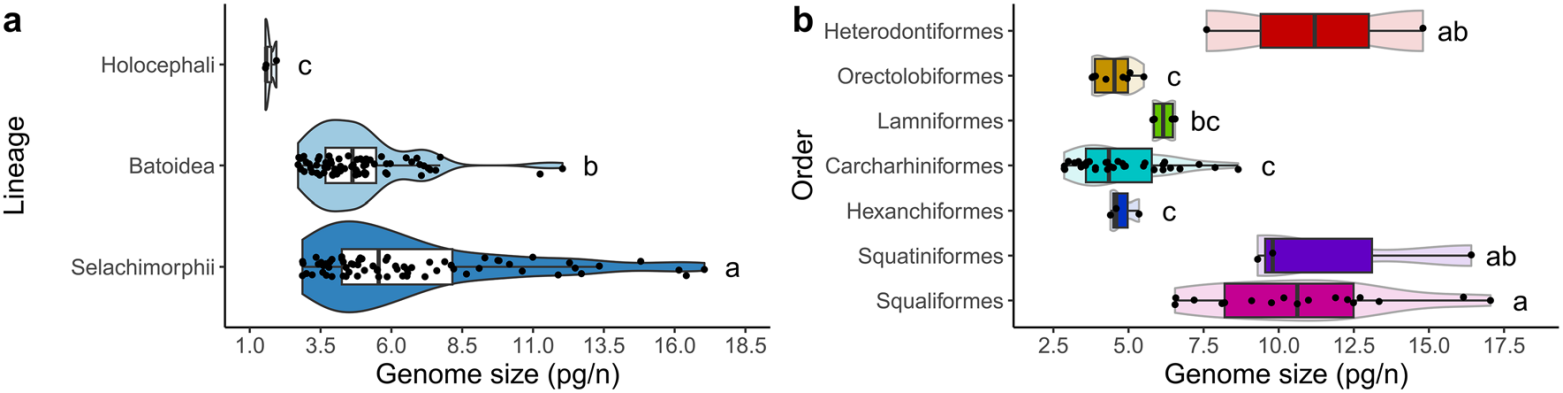
Genome size diversity in Chondrichthyes. (**a**) Distribution of available C-value information (haploid DNA content in picograms, pg/n) across the three major chondrichthyan lineages. (**b**) Distribution of C-values across the shark orders for which genome size data are available (summarized in Table 1). For (**a**, **b)** individual jitter points depict average values per species. Boxes marked with different letters (a, b, or c) were significantly different from one another in post hoc pairwise comparisons, after ln-transformation (i.e., *p* < 0.05). Note the different scale limits for each plot.

Average genome size estimates (C-values) were obtained for a total of 142 chondrichthyan species: 71 sharks, 68 batoids, and 3 chimaeras. Analysis of variance showed significant differences among major chondrichthyan lineages (ANOVA: *F*_(2,139)_ = 19.04, *p* < 0.001***), with sharks having significantly larger genomes (mean ± SD = 6.72 pg ± 3.53) than both batoids (4.86 pg ± 1.79; *t-test* = 3.83, *p* < 0.001***) and chimaeras (1.69 pg ± 0.22; *t-test* = 5.33, *p* < 0.001***) and batoid genomes being significantly larger than those of chimaeras (*t-test* = 4.22, *p* < 0.001***) in post hoc (Tukey’s HSD) analysis (Fig. 1a). Within sharks, interspecific C-value variation ranged about sixfold, with the highest values assigned to the Squalomorphii clade in comparisons across superorders (ANOVA: *F*_(1,68)_ = 74.51, *p* < 0.001***). Shark orders also differed significantly from one another in average DNA content (ANOVA: *F*_(6,64)_ = 22.57, *p* < 0.001***), with post hoc analysis grouping Heterodontiformes, Squatiniformes and Squaliformes (accounting for the largest genomes) separated from Orectolobiformes, Carcharhiniformes and Hexanchiformes. With intermediate C-values, Lamniformes were only significantly different from Squaliformes (Table 1, Fig. 1b).

**Table 1:** Summary statistics of haploid genome size and karyotype parameters across shark clades.

Classification	Genome size (pg/n)				Chromosome number (n)				Fundamental number (FN)			
	*n*	Range	Mean ± SD	CV	*n*	Range	Mean ± SD	CV	*n*	Range	Mean ± SD	CV
**Selachimorphii** (or Selachii)	71	2.86–17.05	6.72 ± 3.53	52.52	36	30.5^a^–54	41.32 ± 7.37	17.82	36	39–80.5^a^	56.94 ± 9.61	16.88
Galeomorphii	48	2.86–14.80	5.08 ± 2.02	39.68	27	31–54	41.74 ± 7.16	17.16	27	45–80.5	59.37 ± 8.97	15.12
Heterodontiformes	2	7.60–14.80	11.20 ± 5.09	45.46	2	51	51 ± 0	0	2	56–64	60 ± 5.66	9.43
Orectolobiformes	8	3.79–5.51	4.51 ± 0.66	14.53	5	51–54	52.2 ± 1.30	2.50	5	59–80.5^a^	70.5 ± 10.01	14.20
Lamniformes	4	5.81–6.55	6.17 ± 0.39	6.27	2	41–42	41.5 ± 0.71	1.70	2	65–66	65.5 ± 0.71	1.08
Carcharhiniformes	34	2.86–8.65	4.73 ± 1.51	31.96	18	31–45	37.83 ± 4.42	11.69	18	45–64	55.53 ± 6.41	11.55
Squalomorphii	23	4.40–17.05	10.13 ± 3.60	35.53	9	30.5^a^–52	40.06 ± 8.26	20.63	9	39–59	49.67 ± 7.95	16.01
Hexanchiformes	3	4.40–5.35	4.78 ± 0.50	10.52	3	36–52	46 ± 8.72	18.95	3	39–56	49.67 ± 9.29	18.71
Squatiniformes	3	9.30–16.41	11.84 ± 3.97	33.53	1	44	44	–	1	57	57	–
Squaliformes	17	6.55–17.05	10.78 ± 3.08	28.55	5	30.5^a^–43	35.70 ± 6.67	18.68	5	41–59	48.20 ± 820	17.01

*CV* Coefficient of variation.

^a^Decimal karyotype number due to averaging for species with multiple entries. See Supplementary Table S1 for species-specific estimates.

### Mode and rate of genome size evolution

**Figure 2:**
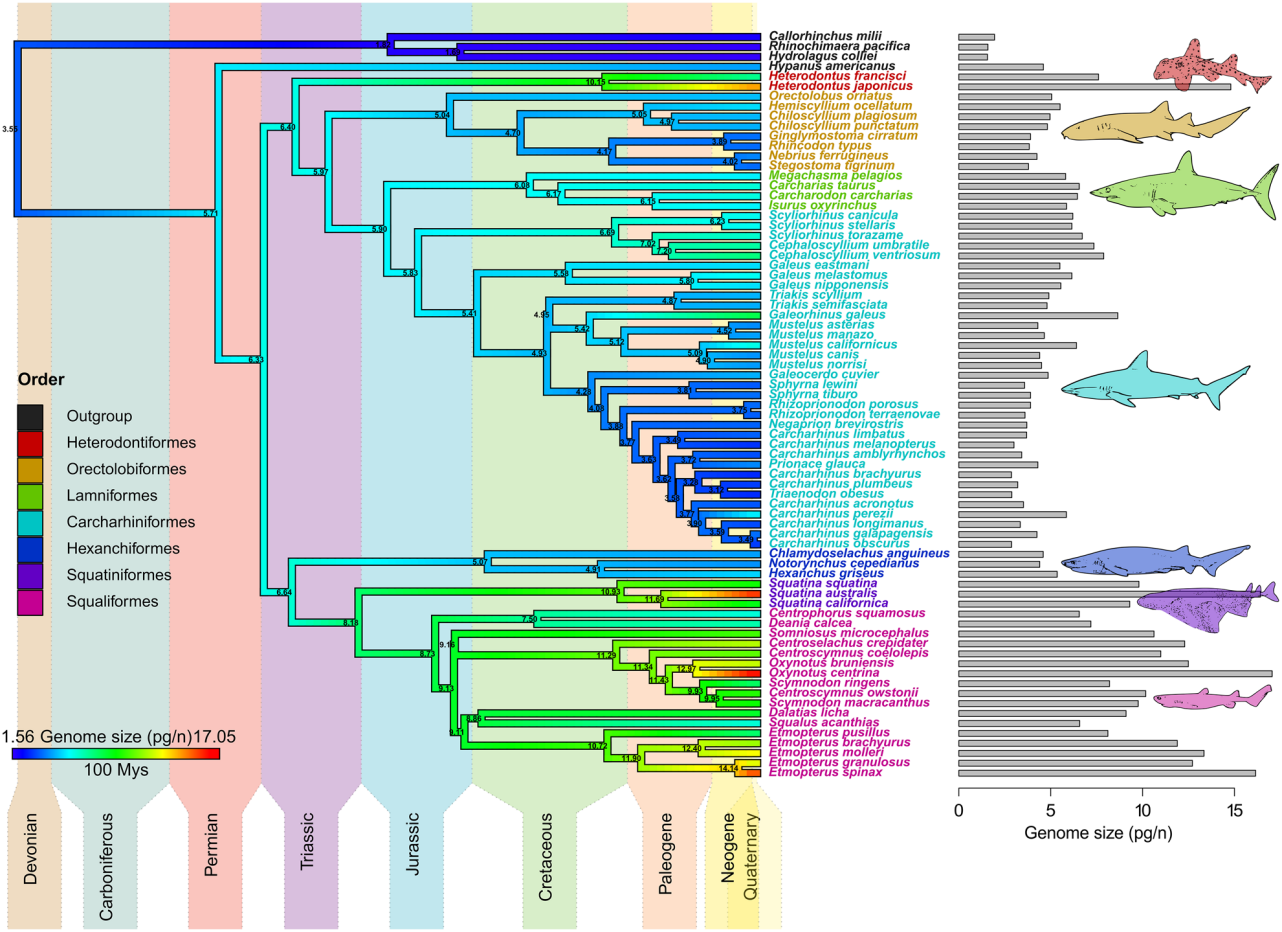
Genome size evolution in sharks and outgroups. Species-level phylogeny (‘Maximum Clade Credibility’ or MCC tree hypothesis) depicting ancestral genome size reconstruction under the best-fitting maximum-likelihood ‘Early-Bust’ (EB) model, with details on geological timescale. Tips are colour-coded, as stated in the inset (bottom-left), by the average genome size of each species (represented in the barplot, right). Internal node colours are based on the most likely ancestral genome size estimates inferred by the model (values reported to two decimal places at nodes). Species names at tips are colour-coded according to the taxonomic order to which they belong, following Fig. 1b colour scheme, with outgroup species in black (left inset). The ancestral genome size state for all extant sharks was estimated at 6.33 pg, and for all extant Chondrichthyes at 3.55 pg. Within Selachii, genome size is differently evolving across higher-level taxa: while galeomorph sharks show a general trend in genome size diminution (with the exception of Heterodontiformes and Scyliorhinidae), most squalomorph sharks (especially Squatiniformes and Squaliformes) exhibit steady genome size increases.

From the three models of evolution fitted to the genome size data (71 shark and 4 outgroup species; Supplementary Table S1), the ‘Early-Burst’ (EB) model was the best-fitting one (AICw = 0.84, with a better fit for the constant-rate ‘Brownian Motion’ (BM) model in analysis lacking an outgroup, AICw = 0.50; more details in Supplementary Table S2). Reconstruction of ancestral values (Fig. 2) showed labile changes in DNA content at different taxonomic levels, with a major rate of increase preceding shark diversification (see Supplementary Fig. S4 for a projection of the phylogeny onto genome size phenotypic space). From this point, larger and smaller genomes were secondarily acquired from an ancestral state for all major extant shark clades (6.33 pg) similar to the present-day average (6.72 pg).

Pagel’s tree transformation parameters were estimated to further test for deviations from a constant-rate process of evolution. Pagel’s lambda (λ) was greater than 0.96, whether an outgroup was included or not (for both cases: *p*_against λ=0_ < 0.001***, *p*_against λ=1_ > 0.598), indicating that phylogeny strongly determined genome size evolution (i.e., strong phylogenetic signal, λ = 1). Pagel’s delta (δ) for all species was 0.21 (*p*_against δ=1_ = 0.018*), characteristic of an evolutionary scenario where character change is concentrated early in the species diversification (i.e., δ < 1). Exclusion of the outgroup species resulted in a higher estimate not significantly different from a situation where the rate of evolution remained constant through time (δ = 0.61, *p*_against δ=1_ = 0.348). The estimates obtained for Pagel’s kappa (κ) were 0.91 (with outgroup, *p*_against κ=1_ = 0.799) and 0.59 (sharks only, *p*_against κ=1_ = 0.304), supporting a pattern of gradual evolution (i.e., κ = 1). For this parameter, the null hypothesis of punctuated evolution could only be rejected when the outgroup was included (*p*_against κ=0_ = 0.025*; sharks only: *p*_against κ=0_ = 0.304).

Finally, the rates at which DNA content evolved across the two shark superorders were compared. Phylogenetic Independent Contrast (PIC) absolute values, which encapsulate the degree of trait change, were on average greater in Squalomorphii (mean ± SD = 0.28 ± 0.24) than in Galeomorphii (0.11 ± 0.15; Mann–Whitney U test: *W* = 238, *p* < 0.001***, *n* = 69). Results for all the analyses described in “Mode and rate of genome size evolution” section were consistent across the 100 alternative phylogenetic hypotheses considered, except for Pagel’s κ concerning sharks only, where estimates widely ranged between 0 and 1 (Supplementary Fig. S5, Table S3).

### Chromosome number evolution

**Figure 3:**
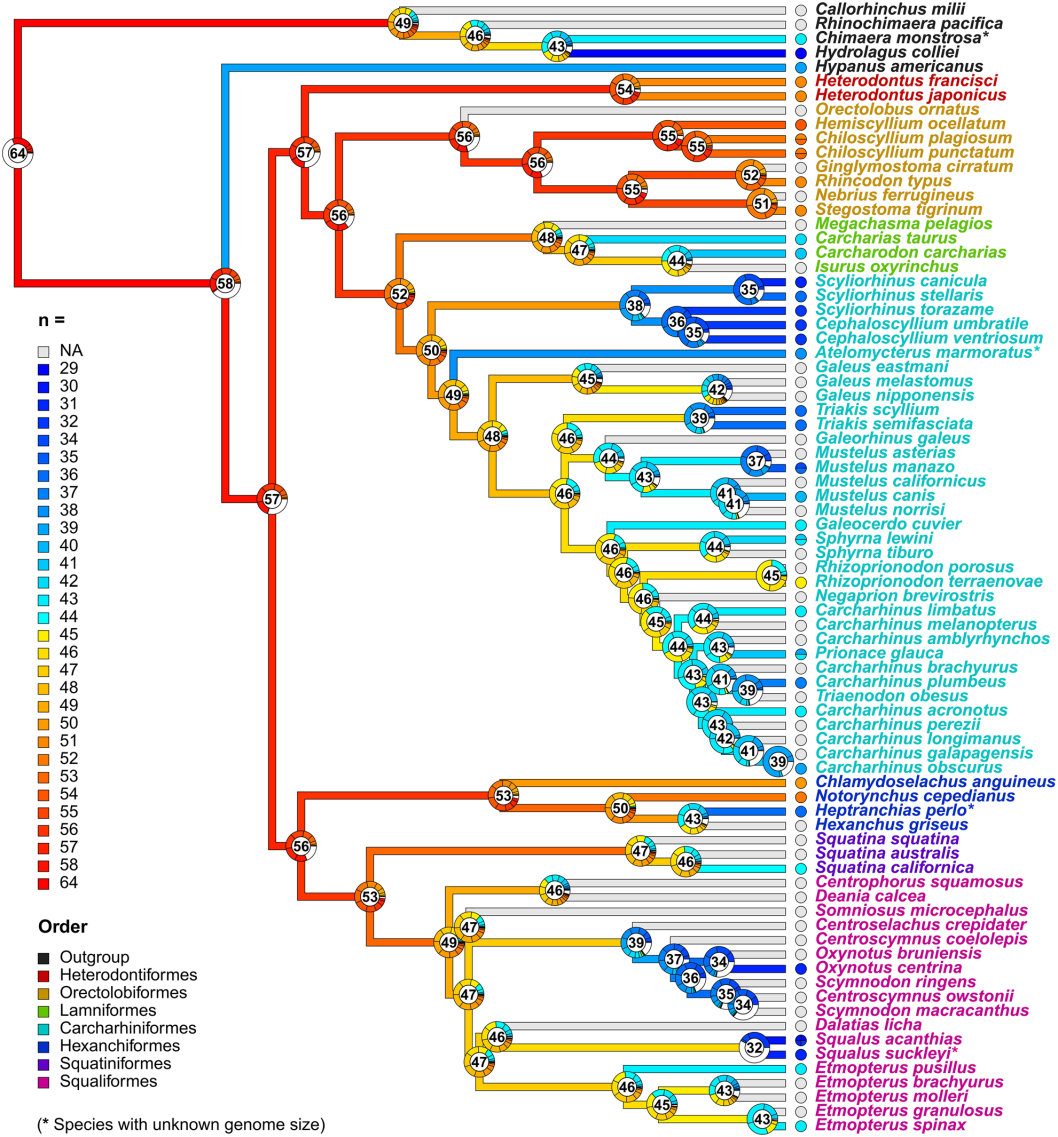
Chromosome number evolution in sharks and outgroups. Species-level phylogeny (MCC tree) depicting ancestral haploid chromosome number (n) reconstruction under the best-fitting maximum-likelihood ChromEvol model (“Constant Rate with No Duplication”). Terminal branches are colour-coded, as specified in the inset (left), by the average chromosome count reported for each species (with details on polymorphic states in the pie charts at tips, species from Fig. 2 with unknown karyotype (NA) coloured in grey, and additional species lacking genome size information—four—marked with “*”). Pie charts at nodes represent the probabilities of the most likely chromosome numbers to exist at any internal node inferred across 10,000 simulations (those included in the inset) out of the summed probabilities of any other chromosome number allowed in the model (in white, see Methods). Numbers inside pie charts correspond to the chromosome numbers inferred with the highest probability. Species names at tips are colour-coded after taxonomic order (bottom-left inset). The reconstruction of ancestral chromosome numbers revealed high number estimates for the common ancestor of all extant Chondrichthyes (n = 64) and sharks (n = 57). In Selachii, the oldest evolutionary lineages within both the Galeomorphii (i.e., Heterodontiformes and Orectolobiformes) and Squalomorphii (i.e., Hexanchiformes) clades maintained similar values (n = 53–56) at their root nodes, approximating the ancestral state estimated for all sharks. Throughout the tree, the primary mechanism of chromosome number change identified was descending disploidy (chromosome loss), particularly evident during the diversification of Carcharhiniformes and Squaliformes.

Ancestral haploid chromosome number (n) reconstruction was based on chromosome count data for 36 shark species (summarized by order in Table 1 and by species in Supplementary Table S1) and 3 outgroup species. From the eight models tested in ChromEvol, “Constant Rate with No Duplication” provided the best fit (AIC = 244.20; more details in Supplementary Table S4). According to this result, only events of dysploidization (i.e., structural chromosome rearrangements) accounted for changes in chromosome numbers along the branches of the tree and thus, no single polyploidization or demi-duplication event was inferred from the analysis (Fig. 3). Overall, inferred occurrences of descending disploidy (i.e., individual chromosome losses through for example fusion events) were more common than those involving ascending disploidy (via fission events): loss rate, δ = 42.10, total frequency = 964.43; gain rate, λ = 23.20, total frequency = 533.56.

**Figure 4:**
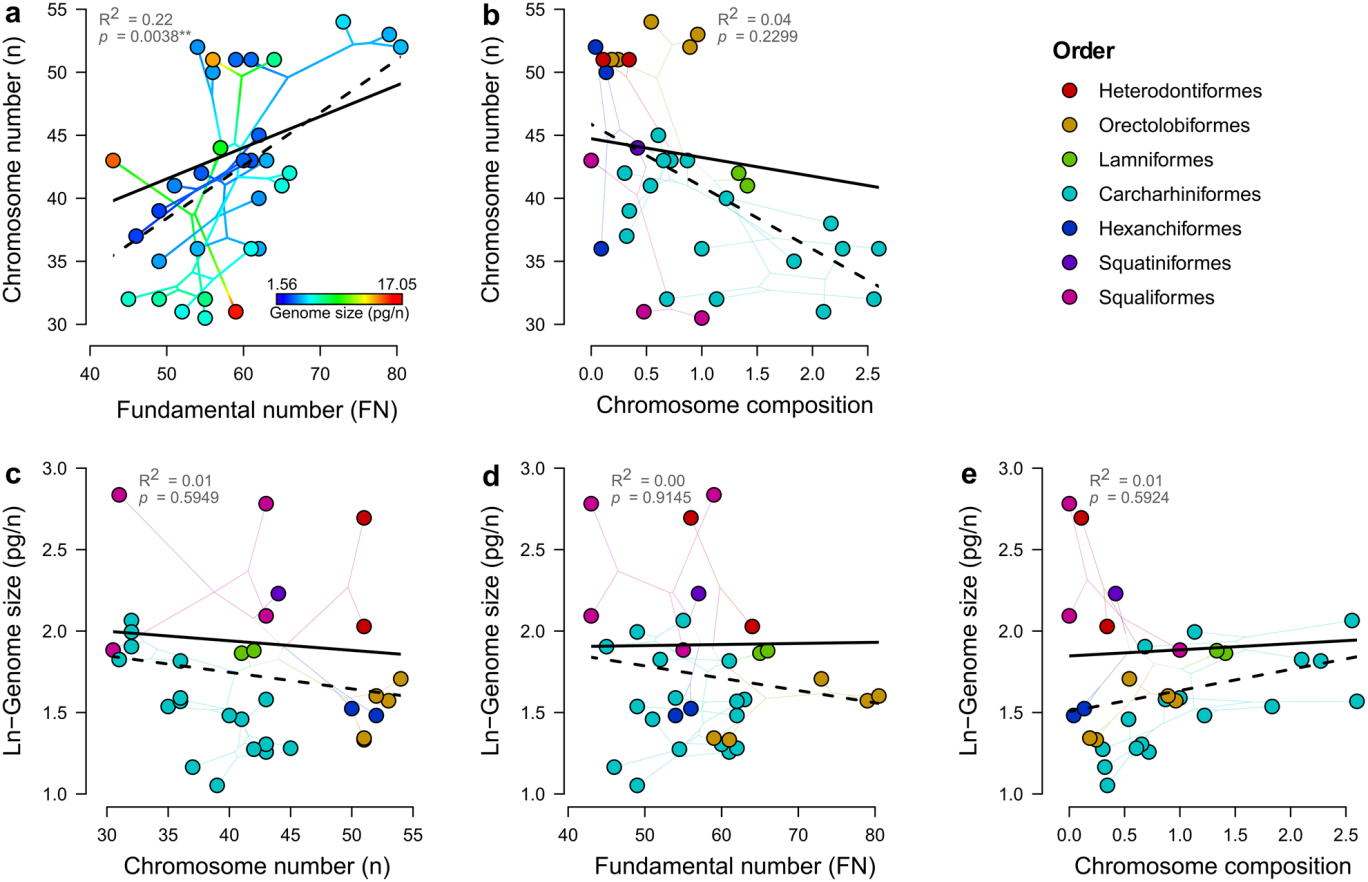
Relationships among karyotype parameters and with genome size in sharks. Species mean values for haploid chromosome number (n) plotted (phylomorphospace) as a function of (**a**) haploid fundamental number (FN) of chromosome arms; and (**b**) chromosome composition. Species mean values for ln-transformed genome size plotted (phylomorphospace) as a function of (**c**) haploid chromosome number (n); (**d**) haploid fundamental number (FN) of chromosome arms; and (**e**) chromosome composition. Lines connecting dots indicate phylogenetic relationships. Solid black lines represent PGLS regression lines, while dashed black lines represent non-phylogenetically corrected (OLS) regression lines (see Supplementary Table S5). For (**a**) dots and internal branches are colour-coded following Fig. 2 ancestral genome size reconstruction, restricted to the taxa for which there is karyotype information. The absence of a colour gradient along n or FN already indicates the lack of association between genome size and any of the two karyotype parameters (as shown in **c** and **d**). For (**b**–**e**), dots and branches are colour-coded after taxonomic order (top-right inset).

Haploid chromosome number (n) was positively correlated with fundamental number (FN) of chromosome arms (Phylogenetic Generalized Least Squares (PGLS): *β* = 0.25, *p* < 0.01**, *df* = 34), but not significantly associated with chromosome composition (i.e., the ratio between two-armed and one-armed chromosome morphotypes; PGLS: *β* = − 1.49, *p* = 0.229, *df* = 33; Fig. 4a,b). No significant associations were found between genome size and any of the karyotype parameters (see details in Table 2, Fig. 4c–e).

**Table 2:** Summary statistics of simple PGLS regression analysis (i.e., not fully-combined or derived models) comparing karyotypic, cytological, life-history, and ecological parameters against (ln-transformed) genome size.

Category	Model predictor(s)	*df*	λ	Slope (*β*) ± SE	*t*-value	*F*-value	*p*-value
Karyotypic and cytological factors	n	31	1 (1)	− 0.006 ± 0.505 (− 0.006)	− 0.537 (− 0559)	0.289 (0.312)	0.5949 (100%)
	FN	31	1 (1)	0.0007 ± 0.006 (− 0.0001)	0.108 (− 0.023)	0.012 (0.014)	0.9145 (100%)
	Chr. comp	30	1 (1)	0.038 ± 0.069 (0.035)	0.541 (0.499)	0.293 (0.249)	0.5924 (100%)
	ln-*C*_*a*_	**31**	**1 (0.96)**	**0.521 ± 0.141 (0.566)**	**3.700 (3.952)**	**13.692 (15.618)**	8.34e−4*** (100%)
	ln-*N*_*a*_	**22**	**∼ 0 (∼ 0)**	**0.779 ± 0.080 (0.788)**	**9.697 (9.675)**	**94.033 (93.609)**	2.11e−09*** (100%)
Morphological factors	*L*_*PCA1*_ + (*L*_*PCA1*_)^2^	67	0.86 (0.84)	*L*: − 0.038 ± 0.032 (− 0.047) *L*^2^: 0.010 ± 0.009 (0.012)	*L*: − 1.183 (− 1.410) *L*^2^: 1.125 (1.312)	*L*: 1.400 (1.989) *L*^2^: 1.266 (1.722)	*L*: 0.2510 (100%) *L*^2^: 0.2646 (100%)
	ln-*W*_*max*_	35	0.88 (0.86)	− 0.014 ± 0.021 (− 0.019)	− 0.667 (− 0.809)	0.445 (0.654)	0.5090 (100%)
	*PBS*	**60**	**0.74 (0.73)**	–	–	**3.086 (3.086)**	0.0223* (98%)
	CFAR*^§^	59	0.83 (0.89)	− 0.050 ± 0.048 (− 0.066)	− 1.054 (− 1.439)	1.111 (2.070)	0.2962 (59%)
Metabolic and physiological factors	ln-*SMR*^§^	**14**	**0 (0)**	− **0.685 ± 0.103 (**− **0.685)**	− **6.356 (**− **6.656)**	**44.300 (44.300)**	1.09e−05*** (100%)
	*L*_*PCA1*_ + *TS* (= Cruising speed)	**62**	**0.77 (0.72)**	*L*: − 0.002 ± 0.023 (− 0.002) ***TS*: –**	*L*: − 0.083 (− 0.103) ***TS*: –**	*L*: 0.007 (0.1010) ***TS*: 3.580 (3.869)**	*L*: 0.9344 (100%) *TS*: 0.0187* (100%)
Developmental and demographic factors	k*^§^	54	0.77 (0.80)	− 0.144 ± 0.551 (− 0.094)	− 0.261 (− 0.173)	0.068 (0.030)	0.7950 (100%)
	*T*_PCA1_*^§^	53	0.84 (0.86)	− 0.087 ± 0.073 (− 0.083)	− 1.203 (− 1.158)	1.447 (1.342)	0.2344 (100%)
	*r*_*max*_^§^	30	0.88 (0.89)	0.244 ± 0.303 (0.319)	0.804 (1.237)	0.647 (1.124)	0.4275 (100%)
Reproductive factors	ln-*L*_*s*_ + *RM*^§§^	64	0.86 (0.84)	*L*_*s*_: 0.006 ± 0.037 (0.003) *RM*: –	*L*_*s*_: 0.268 (0.083) *RM*: –	*L*_*s*_: 0.027 (0.009) *RM*: 2.111 (2.241)	*L*_*s*_: 0.8710 (100%) *RM*: 0.1295 (100%)
Ecological factors	Temp	68	0.89 (0.88)	0.002 ± 0.006 (0.001)	0.323 (0.222)	0.104 (0.072)	0.7479 (100%)
	ln-Depth	69	0.89 (0.87)	− 0.009 ± 0.030 (− 0.006)	− 0.309 (− 0.198)	0.095 (0.060)	0.7586 (100%)
	ln-Depth range	69	0.89 (0.87)	− 0.006 ± 0.030 (− 0.010)	− 0.195 (− 0.324)	0.038 (0.105)	0.8463 (100%)
	ln-Depth + Climate^†^	65	0.88 (0.85)	Dep: − 0.004 ± 0.031 (0.001) Clim: –	Dep: − 0.131 (0.046) Clim: –	Dep: 0.017 (0.018) Clim: 0.192 (0.269)	Dep: 0.8959 (98%) Clim: 0.9417 (98%)
	ln-Depth + Occurrence^†^	67	0.89 (0.87)	Dep: − 0.007 ± 0.032 (0.004) Oc: –	Dep: − 0.229 (0.123) Oc: –	Dep: 0.053 (0.040) Oc: 0.057 (0.076)	Dep: 0.8193 (100%) Oc: 0.9446 (100%)
	Habitat	67	0.88 (0.87)	–	–	0.652 (0.775)	0.5846 (100%)
	ln-Depth + Salinity^†^	**67**	**0.91 (0.91)**	Dep: − 0.019 ± 0.030 (− 0.024) **Sal: –**	Dep: − 0.668 (− 0.816) **Sal: –**	Dep: 0.446 (0.666) **Sal: 5.187 (6.002)**	Dep: 0.5066 (96%) Sal: 0.0081** (100%)

Each test statistic was calculated using the ‘Maximum Clade Credibility’ (MCC) tree as the main phylogenetic hypothesis and is followed (in parenthesis) by the median value calculated over the 100 alternative trees. For the *p*-value, the number in parenthesis refers to the percentage of trees with a *p*-value falling on the same side of the significance threshold (α = 0.05) as for the MCC tree (significant results in bold: *p ≤ 0.05; **p ≤ 0.01; ***p ≤ 0.001). Regarding categorical predictors, parameter effects were only summarized using type II sum of squares for simplicity (*F*-test; with details on post hoc pairwise comparisons available in Supplementary Tables S7–S9). Note that in models containing more than one predictor, interaction terms rendered non-significant results and thus, were excluded from the final analysis (abbreviations, n: Chromosome number; FN: Fundamental number of chromosome arms; Chr. comp.: Chromosome composition; *C*_*a*_: Cell area; *N*_*a*_: Nucleus area; *L*_*PCA1*_ (or *L*): Total body length (PCA1 scores); *W*_*max*_: Maximum body weight; *PBS*: Precaudal body shape; *CFAR*: Caudal fin aspect ratio; *SMR*: Standard metabolic rate; *TS*: Tail shape; *k*: Growth completion rate; *T*_*PCA1*_: Age (PCA1 scores); *r*_*max*_: Maximum intrinsic rate of population increase; *L*_*s*_: Litter size; *RM*: Reproduction mode; Temp: Preferred water temperature; Depth (or Dep): Average depth; Clim: Climate; Oc: Occurrence; Sal: Salinity). Detailed definitions for each biological parameter are available in Supplementary Methods.

*Additional results for when poor-quality estimates were omitted (see Supplementary Methods) are shown in the main text.

^§^Body-size correction using regression residuals (i.e., partial correlation analysis).

^§§^Additional body-size corrected results (via multiple regression) are shown in the main text.

^†^Depth correction performed by including ln-tranformed average depth (“ln-Depth”) as a covariate (i.e., ANCOVA).

### Genome size variation across cytological, life-history, and ecological gradients

#### Simple regression analysis

Genome size variation was compared against a wide variety of phenotypic and ecological parameters via PGLS, summarized in Table 2. Further details, including non-phylogenetically corrected (OLS) regression results, post hoc pairwise comparisons, and visualizations are available in Supplementary Tables S5, S7–S9, and Figs. S6–S9.

Within the cell, a strong positive relationship was found between genome size and both erythrocyte cell (*C*_*a*_) and nucleus (*N*_*a*_) areas. Regarding morphological factors, neither total body length (*L*_*PCA1*_) nor maximum body weight (*W*_*max*_) were significantly associated with genome content. Precaudal body shape (*PBS*), on the other hand, was a better predictor of genome size. Post hoc analysis revealed that species with body types 4 and 5 exhibit larger genomes than those embodying more active lifestyles (body types 2 and 3), while sharks with body type 1 were not statistically different from any of them (for body type definitions, see Supplementary Methods). Body-size corrected caudal fin aspect ratio (*CFAR*, a general indicator of swimming activity) was significantly negatively correlated with genome size only when poor-quality values (i.e., estimated from illustrations) were omitted from the analysis (partial PGLS correlation (PC_PGLS_): *β* = − 0.12, *p* < 0.001***, *df* = 37).

Among physiological predictors, (body-mass corrected) standard metabolic rate (*SMR*) was strongly negatively associated with DNA content. At the functional level, analysis of genome size covariance across (body-size corrected) tail shapes (*TS*), which relate to the species’ cruising speed of locomotion, revealed significant differences: tail type 4 species, characterized by the slowest cruising speeds, contained significantly higher C-values than those with faster locomotion mechanics (tail types 2 and 3). Tail type 1 species (with the second fastest cruising speeds) were not statistically different from any group (tail type definitions are given in Supplementary Methods).

With respect to developmental and demographic traits, there were no significant effects of (body-size corrected) growth completion rate (*k*), age (*T*_*PCA1*_), or maximum intrinsic rate of population increase (*r*_*max*_) on DNA content. Discarding *k* and *T*_*PCA1*_ poor-quality estimates (i.e., those calculated through the FishBase Life-history tool) led to similar results (PC_PGLS_, *k*: *β* = 0.19, *p* = 0.696, *df* = 40; *T*_*PCA1*_: *β* = − 0.07, *p* = 0.224, *df* = 39).

Among reproductive parameters, no significant relationships were found between genome size and reproduction mode or the covariate litter size (*L*_*s*_). Factoring out the effects of body size rendered almost identical results (multiple PGLS regression (MR_PGLS_), reproduction mode: *F*_(2,64)_ = 2.12, *p* = 0.128; *L*_*s*_: *β* = 0.03, *p* = 0.500; *df* = 63 for both).

In relation to the ecological factors analysed, no significant associations were found between DNA content and preferred water temperature, average depth, or depth range. Similarly, environmental differences in genome size according to (depth corrected) climate, species occurrence across the water column, and habitat (not corrected by water-depth) were not significant, unlike for water salinity: sharks inhabiting marine waters had genomes significantly larger than those frequenting marine-brackish waters and species capable of entering rivers (here, amphidromous).

#### Multivariate regression analysis

Based on AICc minimization, the best-supported combination of life-history and ecological predictors explaining shark genome size variation is given in Table 3. Further details, including post hoc pairwise comparisons for categorical predictors, are available in Supplementary Tables S6–S10.

**Table 3:** Assessment of the best minimum adequate multivariate PGLS models based on AICc minimization (i.e., models with AICc_i_ − AICc_min_ < 2).

Full model predictors	Best models	*df*	log-lik	AICc	*F*-value	*p*-value
(1) *L*_*PCA1*_ + (*PBS or* Oc) + *RM* + ln-*L*_*s*_ + (Temp *or* ln-Depth *or* ln-Depth range) + Clim + Sal (n = 62)	(1.1) 1 + *L*_*PCA1*_ + *PBS* + *RM* + ln-*L*_*s*_ + Sal	11	16.34	− 5.41	*L*_*PCA1*_: 0.079 (0.069) ***PBS*: 5.530 (5.245)** ***RM*: 3.948 (3.818)** ln-*L*_*s*_: 3.245 (3.313) **Sal: 7.338 (7.679)**	0.7801 (100%) 0.0009*** (100%) 0.0255* (96%) 0.0776 (100%) 0.0016** (100%)
	(1.2) 1 + *L*_*PCA1*_ + *PBS* + *RM* + Sal	10	14.44	− 4.56	*L*_*PCA1*_: 0.111 (0.131) ***PBS*: 4.855 (4.461)** ***RM*: 3.234 (3.092)** **Sal: 5.838 (6.237)**	0.7402 (100%) 0.0021** (100%) 0.0475* (42%) 0.0052** (100%)
	(1.3) 1 + *L*_*PCA1*_ + *PBS* + ln-*L*_*s*_ + Sal	9	13.01	− 4.56	*L*_*PCA1*_: 0.008 (0.010) ***PBS*: 3.026 (2.825)** ln-*L*_*s*_: 2.474 (2.465) **Sal: 7.965 (8.770)**	0.9312 (100%) 0.0254* (80%) 0.1217 (99%) 0.0009*** (100%)
	(1.4) 1 + *L*_*PCA1*_ + *PBS* + Sal	8	11.60	− 4.48	*L*_*PCA1*_: 0.574 (0.589) ***PBS*: 3.224 (2.925)** **Sal: 6.738 (7.578)**	0.4518 (100%) 0.0191* (87%) 0.0024** (100%)
	(1.5) 1 + *L*_*PCA1*_ + *PBS* + *RM* + ln-*L*_*s*_ + ln-Depth + Sal	12	17.38	− 4.39	*L*_*PCA1*_: 0.018 (0.013) ***PBS*: 6.592 (6.080)** ***RM*: 4.667 (4.476)** **ln-*L***_***s***_**: 4.120 (4.191)** ln-Depth: 1.718 (1.659) **Sal: 8.226 (8.489)**	0.8938 (100%) 0.0002*** (100%) 0.0139* (100%) 0.0477* (66%) 0.1960 (100%) 0.0008*** (100%)
	(1.6) 1 + *L*_*PCA1*_ + ln-*L*_*s*_ + Sal	5	7.68	− 4.29	*L*_*PCA1*_: 0.039 (0.033) ln-*L*_*s*_: 3.007 (2.810) **Sal: 8.006 (9.310)**	0.8433 (100%) 0.0883 (95%) 0.0009*** (100%)
	(1.7) 1 + *L*_*PCA1*_ + Sal	4	6.09	− 3.48	*L*_*PCA1*_: 0.337 (0.312) **Sal: 6.680 (8.024)**	0.5638 (100%) 0.0025** (100%)
(2) *L*_*PCA1*_ + (*PBS or* Hab) + *RM* + ln-*L*_*s*_ + (Temp *or* ln-Depth *or* ln-Depth range) + Clim + Sal (n = 62)	As in models 1.1 to 1.7					
(3) *L*_*PCA1*_ + (*TS or* Oc) + *RM* + ln-*L*_*s*_ + (Temp *or* ln-Depth *or* ln-Depth range) + Clim + Sal (n = 63)	(3.1) 1 + *L*_*PCA1*_ + *TS* + Sal	7	8.12	− 0.20	*L*_*PCA1*_: 0.186 (0.170) ***TS*: 3.450 (3.403)** **Sal: 5.883 (6.319)**	0.6679 (100%) 0.0225* (98%) 0.0048** (100%)
	(3.2) 1 + *L*_*PCA1*_ + *TS* + *RM* + Sal	9	10.69	0.02	*L*_*PCA1*_: ~ 0 (0.009) ***TS*: 4.931 (5.076)** *RM*: 2.697 (2.807) **Sal: 4.863 (5.163)**	0.9834 (100%) 0.0042** (100%) 0.0765 (64%) 0.0114* (100%)
	(3.3) 1 + *L*_*PCA1*_ + *TS* + ln-*L*_*s*_ + Sal	8	8.83	1.01	*L*_*PCA1*_: ~ 0 (0.005) ***TS*: 3.520 (3.544)** ln-*L*_*s*_: 1.255 (1.318) **Sal: 6.490 (6.930)**	0.9855 (100%) 0.0208* (99%) 0.2674 (100%) 0.0029** (100%)
	(3.4) 1 + *L*_*PCA1*_ + *TS* + *RM* + ln-*L*_*s*_ + Sal	10	11.59	1.05	*L*_*PCA1*_: 0.194 (0.232) ***TS*: 5.568 (5.801)** *RM*: 2.993 (3.114) ln-*L*_*s*_: 1.564 (1.700) **Sal: 5.580 (6.111)**	0.6618 (100%) 0.0021** (100%) 0.0587 (54%) 0.2166 (100%) 0.0063** (100%)
	(3.5) 1 + *L*_*PCA1*_ + Sal	4	3.58	1.53	*L*_*PCA1*_: 0.093 (0.052) **Sal: 6.188 (7.021)**	0.7616 (100%) 0.0036** (100%)
(4) *L*_*PCA1*_ + (*TS or* Hab) + *RM* + ln-*L*_*s*_ + (Temp *or* ln-Depth *or* ln-Depth range) + Clim + Sal (n = 63)	As in models 3.1 to 3.5					

This stepwise model selection procedure was based on the MCC phylogenetic hypothesis. For the *F*-value, median values calculated over the 100 alternative trees are given in parenthesis. For the *p*-value, the number in parenthesis refers to the percentage of trees with a *p*-value falling on the same side of the significance threshold (α = 0.05) as for the MCC tree (significant results in bold: *p ≤ 0.05; **p ≤ 0.01; ***p ≤ 0.001; abbreviations as in Table 2). Different full models (models 1 to 4) were used to analyse predictors with aliased coefficients separately (i.e., *PBS* and *TS,* occurrence and habitat), where predictors not allowed to co-occur during model selection (see Methods) are connected by “*or*”. Among all life-history and ecological parameters, the variables *W*_*max*_, *CFAR*, *SMR*, *k*, *T*_*PCA1*_, and *r*_*max*_ were excluded from the analysis given their scattered availability, while *L*_*PCA1*_ was set as a fixed covariate during all permutations to control for allometry. Details regarding post hoc pairwise comparisons for categorical predictors are available in Supplementary Tables S7–S10.

The predictors *L*_*s*_, preferred water temperature, average depth, depth range, climate, and occurrence were excluded from the best-supported multivariate models or, when included, were not significantly associated with genome size (in agreement with “Simple regression analysis” section). Similarly, results were consistent with “Simple regression analysis” section for *L*_*PCA1*_, *PBS* (with the difference between body types 1 and 5 now being significant, *p* = 0.019*), *TS*, and salinity in all top-ranking models. In contrast, reproduction mode had significant effects on genome size in 3 of the 5 best models where it was included. Post hoc comparisons revealed that placental viviparous species harbour genomes significantly smaller than those exhibiting aplacental viviparous and oviparous reproduction.

### The interplay between genome size and inter- and intra-specific diversity

A comparison was made between DNA content and diversity at the taxonomic and genetic levels. No significant associations were found with any of the taxonomic diversity parameters, namely the number of species and genera per family (PGLS: *β* = 0.003, *p* = 0.945; and *β* = − 0.050, *p* = 0.340; *df* = 69 for both; respectively) and the number of species, genera, and families per order (PGLS: *β* = − 0.006, *p* = 0.927; *β* = − 0.092, *p* = 0.187; and *β* = − 0.175, *p* = 0.133; *df* = 69 for all; respectively); nor with expected heterozygosity (*H*_*e*_, PGLS: *β* = − 0.64, *p* = 0.137, *df* = 18; with almost identical results when correcting for body size and age, MR_PGLS_: *β* = − 0.65, *p* = 0.163, *df* = 16; Supplementary Fig. S10). Results for all the comparative analyses described in “Chromosome number evolution”, “Genome size variation across cytological, life-history, and ecological gradients” and “The interplay between genome size and inter- and intra-specific diversity” sections were robust to phylogenetic uncertainty (Tables 2, 3, and Supplementary Table S5).

## Discussion

Our study has confirmed the existence of large genome size differences not only among the three major chondrichthyan lineages, among which sharks represent the group with the largest genomes^10^, but also within shark clades (Table 1, Fig. 1). The sixfold range of DNA content variation observed is mostly driven by the extraordinarily large genomes of certain species of heterodontiforms, squatiniforms, and squaliforms. Within this variation, similar genome sizes tend to occur among closely related species, as indicated by phylogenetic signal (Pagel’s λ) tests.

Ancestral reconstruction analysis revealed shark genomes to be evolutionarily labile in size (Fig. 2), with high rates of change within the squalomorph lineage. This agrees with the expectation that faster rates of change will occur in larger genomes, given that the magnitude of insertion and deletion mutations (indels) and subsequent effects strongly depend on the preceding state of genome size^68^. The lack of articulated fossils, scarce due to the cartilaginous skeleton of Chondrichthyes, prevented the possibility of identifying a clear trend of evolution (unlike for lungfishes and amphibians, where the use of bone lacunae as an indirect estimate of fossil cell, and hence genome size, showed low values as the general ancestral condition^85,86^). As such, genomic expansions and contractions appeared as secondarily derived in different lineages. Despite this limitation, our analysis recovered an ancestral state for all Chondrichthyes (3.55 pg) falling within the range of values estimated at the baseline for all tetrapods (3.2–3.9 pg)^85^. From the split between Holocephali and Elasmobranchii (∼ 380 Mys ago)^87^, a major genome expansion presumably concentrated in the lineage leading to the common ancestor of extant sharks (∼ 250 Mys ago)^2^ during the late Paleozoic (see Supplementary Fig. S4), as suggested by our best-fitting ‘Early-Burst’ model and low Pagel’s δ estimates (but also by the better fit of a constant-rate model of evolution and higher Pagel’s δ values in analyses lacking an outgroup—comprised mostly by chimaeras).

Regarding the proximate mechanisms underlying this evolutionary trend, no associations were found between genome size and any karyotype parameter (Table 2, Fig. 4c–e), suggesting that chromosome-level alterations are not an important contributor to overall genome size diversity in this group (unlike for ray-finned fishes^27,88^). The gradual evolution of genome size suggested by Pagel’s κ metrics should nevertheless be interpreted with caution, given the lack of consistent results to sample size variation and phylogenetic uncertainty (Supplementary Fig. S5), and the observation of drastic shifts in DNA content between closely related species unconnected to karyotype (Fig. 4a).

From the karyotype profile of extant Chondrichthyes, it has been proposed that a polyploidization event predated the early divergence of elasmobranchs, given the lower arm numbers—and genome sizes—seen in holocephalan species^7,13^. This event was presumed to be followed by successive rediploidization events, with progressive chromosome fusions reducing chromosome number in more recent lineages, given their parallel increase in two-armed chromosomes relative to one-armed ones—here, chromosome composition (the *fusion model* hypothesis)^7,13^. However, no evidence of polyploidization events (i.e., total frequency = 0) was obtained from our best chromosome evolution model (or alternative models; Supplementary Table S4), nor signs of whole-genome duplications unique to sharks (or elasmobranchs) have been detected in recent genome-wide studies^14,15^. While the evolutionary history of genetic scaffolding recovered here was dominated by chromosome fusion events, as previously suggested, these are rather pervasive in vertebrate chromosome differentiation and not necessarily indicative of past rediploidization events^89^. The fact that chromosome number was related to fundamental number (FN) but not to chromosome composition (Fig. 4a,b) suggests that, in addition to chromosome rearrangements, other processes such as aneuploidy may have influenced the karyotype evolution of sharks. Our ancestral reconstruction analysis therefore suggests that a large number of elements characterized the ancestral karyotypic state of all sharks and Chondrichthyes, consistent with the high chromosome numbers of recently-karyotyped orectolobiform sharks^38^ (and other evolutionary-old shark lineages; Fig. 3), and those of reconstructed ancestral proto-gnathostome genomes^90^.

Over the last years, the advent of genomic tools has revealed eukaryote genome size diversity to be mostly driven by differences in the abundance of repetitive elements, especially transposable elements (TEs)^91–93^. Although based on a limited number of species, the compositional characteristics of shark genomes, obtained from old C_0_t analyses^94^ and more recent whole-genome assemblies^5,14^, show the same trend. It seems plausible that differential abundance of TEs is almost certainly the main molecular contributor to genome size diversity also among sharks. Interestingly, as during shark genome evolution, significant expansions in the genomes of fossil lungfishes and lissamphibians took place independently also during the late Paleozoic, which have been hypothesized to be the result of bursts of transposon activity induced by the climatological stress of the time^85^.

With respect to the evolutionary forces shaping the diversity of shark DNA content potentially brought about by TEs, our comparative analyses point at metabolism (but not development) as an important modulator of genome size, as is the case for birds and mammals (Table 2, Supplementary Fig. S6). Not surprisingly, shark erythrocyte cell and nucleus sizes are shown to change in concert with DNA content, a universal relationship in eukaryote cell biology^16^. Species with large genomes (and hence, cells) analysed herein were not characterized by relatively large body sizes. In fact, variations in body size are mostly due to differences in cell number (rather than their size) in most—especially large—vertebrates^10^.

Our study revealed that decreasing shark genome sizes relate to increasing metabolic rates (*SMR*), a pattern not found across other dominantly ectothermic vertebrates species^95,96^, including ray-finned fishes^9,27^. In homeotherms, this relationship has been linked to the higher physiological requirements of active organisms (indirectly) imposing a constraint on genome size evolution through selection for smaller erythrocyte cells^19–21^, as they facilitate gas exchange^18^. This study extends this interpretation to the aquatic realm and provides a mechanistic link, as genome size (which scaled positively with erythrocyte size) assorted according to anatomical features ascribed to swimming ability (i.e., *CFAR*^97,98^, *PBS*^52^, and *TS*^54^; Tables 2, 3) and thus, metabolic rate^99^. In general, strong swimmers (such as the Blue shark, *Prionace glauca*) possess the smallest genomes, while species with slower swimming activities (such as the Greenland shark, *Somniosus microcephalus*) harbour some of the largest; mirroring the patterns observed across comparable flight-efficiency parameters in birds^100,101^. However, the genomes of species with body and tail type 1 (torpedo shape with high aspect-ratio tail), typical of pelagic fast-swimmers, did not show a trend in any direction in most models (Supplementary Tables S7, S8). Interestingly, these were regional endothermic lamniforms, whose intermediate C-values (∼ 6 pg) ranked above those of other pelagic ectothermic relatives (Supplementary Fig. S9 online). It has long been pointed out that the evolutionary forces shaping genome size vary according to the biology of specific clades^10^. The particular thermoregulation of lamniform sharks may have relaxed the selective pressures on keeping their cells and genomes small, as their ability to passively retain metabolic heat from muscle and digestion activity^8^ could result in higher metabolic rates for a given erythrocyte size. Indeed, the endothermic lamniform Mako shark (*Isurus oxyrhinchus*) classified as an outlier in genome-size-*SMR* regressions, despite their middle-sized erythrocytes.

The predictive power of metabolic constraints on genome size did not, however, remain in comparisons across ecotypes linked to different swimming performances (i.e., occurrence). The larger genomes of epibenthic sharks relative to those with more active pelagic and benthopelagic lifestyles were only significant in non-phylogenetically corrected (OLS) analysis and thus, were driven by common ancestry (Supplementary Table S5, Fig. S9). In fact, erythrocyte properties (including size and volume) and genome size were also not associated with these ecological traits—once phylogeny was accounted for—in both elasmobranchs^102^ and ray-fined fishes^27^, respectively.

Comparably, the presumed slower (*k*) and longer (*T*_*PCA1*_) development of sharks with larger genomes (as they incur longer times for cell division and other processes^16^) was only apparent in OLS analysis (Supplementary Table S5, Figs. S6, S7). Significant effects were also not found for the demographic parameter *r*_*max*_, which in turn is dependent on the speed of organismal development^78^. Similarly, the associations reported between *k* and population doubling time with genome size in actinopterygian fishes dissolved in comparisons at higher taxonomic levels^27^. Indeed, this relationship with development is best established within amphibians^95,103^, while seemingly unimportant in most amniotes^96,104^. Longevity, on the other hand, has been repeatedly rejected as a convincing correlate of genome size across all vertebrate groups examined^105^, among which sharks are not an exception. Surprisingly, other than body size, shark adult growth rate seems to be far more related to water temperature than metabolism itself (i.e., temperature-corrected *SMR*; Supplementary Fig. S3), potentially explaining the lack of genome size restrictions linked to development at this life stage. If developmental constraints were to apply on DNA content, they would most notably occur during time-limited stages of intense growth, like metamorphosis in amphibians and insects^103^ or early development in fishes, when cell division rates are paramount. In this regard, placental viviparous sharks exhibited the smallest genomes (Table 3; Supplementary Table S10, Fig. S8). Since time for embryonic growth appears rather constraint across reproduction strategies (average gestation period *vs.* reproduction mode [body- and litter-size corrected], ANCOVA_PGLS_: *F*_(4,42)_ = 1.10, *p* = 0.343), it is possible that placental viviparity implies a relatively more demanding developmental program, for which small genomes are best suited. Indeed, placental viviparous sharks produce larger embryos than oviparous ones of similar size^8^, and the extra-supply of food coming from the placenta may well underlie the higher cell division rates (and numbers) necessary to produce larger offspring in a similar amount of time. Although a tentative explanation, the reasons behind this finding are worthy of further investigation.

At wide ecological scales, DNA content did not show a trend across a climate gradient. This lacking relationship remained across differences in temperature resulting also from water-depth (i.e., preferred water temperature) or depth itself (i.e., average depth, or non-significant differences between deep-sea sharks relative to shallow water inhabitants in comparisons across habitats; at least in phylogenetically corrected analysis; Supplementary Table S5, Figs. S7, S9). These findings contrast with the prediction that large genomes (with reduced metabolism) may be adaptive in frigid settings^23^, given the low energy supply and high oxygen concentrations associated with these environments^24^. Similar scenarios have been reported for actinopterygian fishes, where an effect of thermal regime on genome size was contradictory across studies^9,25^, or non-existent when excluding polyploids or after controlling for taxonomic proximity^27^. Similarly, the claimed increase in genome size with water-depth in argentinoid fishes^26^ turned negligible with denser taxonomic sampling^106^. Thus, this study further supports the lack of selective pressures on genome size coming from temperature (or depth) differences in fishes. In this case, it is possible that the effect of temperature on molecular diffusion rates alone predisposes an organism’s metabolism—particularly in ectotherms—to match the energy availability associated with any given climate (at least to a large degree^24,107^). This explanation would be compatible with the unexpectedly large genomes of active lamniforms, characterized by high energy demands and relatively high body temperatures (in this case from regional endothermy).

Breadth of ecological tolerance, on the other hand, did not have the predicted effect on genome size. Sharks venturing marine-brackish waters (such as the Copper shark, *Carcharhinus brachyurus*) and rivers (such as the Blacktip reef shark, *C. melanopterus*) included species with smaller genomes than those associated with the more stable settings offered by exclusively marine environments (Tables 2, 3; Supplementary Table S9, Fig. S9). The opposite trend has been reported for ray-finned fishes in fresh *versus* marine water comparisons^9,27,28,108^. Interestingly, osmoregulatory energy costs associated with reduced water salinity have been reported for the euryhaline bat ray (*Myliobatis californica*)^109^. Thus, it is possible that changing salinity constrains genome size via its associated metabolic costs rather than by means of ecological amplitude.

The apparent long-term maladaptive consequences of large genome expansions, evidenced by parallel reductions in species richness^29^ and increases in extinction risk^110^ across vertebrates, were not found in the present study (Supplementary Fig. S10). These trends, however, appeared only at high taxonomic levels (above order). This suggests that the threat to lineage survival (if any) from large accumulations of (mostly non-coding) DNA may occur at deeper evolutionary timescales than the diversification of extant sharks. Actually, genome size was not associated with extinction risk *within* Chondrichthyes in previous studies^110^. In contrast, more recent empirical evidence has placed genome expansions at the base of evolutionary radiations in specific taxa, indicating that the particular molecular mechanisms at play can predispose lineages to very different fates^111^. The scarcity of very large genomes observed here (Fig. 1b) may not be necessarily maladaptive. As aforementioned, the rate of change is lower in small genomes, so it is less probable for a small genome to get larger than for a large genome to get smaller, resulting in a skewed distribution toward low C-values^68^.

Finally, the proposition that population size is the dominant factor driving diversity in genome size (due to variable selection efficiency against slightly-deleterious DNA accumulations)^30^ was not supported in the present study (Supplementary Fig. S10). Genome size was not associated with genetic heterozygosity and thus with population size (as suggested by the lack of nucleotypic effects on body size, which in turn scales negatively with population size). However, whether molecular indicators of heterozygosity encapsulate long-term effective population sizes is dubious, leaving this possibility open. Nonetheless, support for this nearly-neutral model of genome size evolution is generally lacking^112^.

## Conclusion

Our study revealed a major expansion in the genomes of late Paleozoic shark predecessors, which was unrelated to the dynamic karyotype evolution of this clade and was most probably accomplished via transposon activity. We also identified sharks as a prime example of metabolic constraints on genome size evolution in ectotherms. The high energy and physiological demands of active swimming behaviours were accompanied by small genome sizes, as were the potentially rapid early developments of placental viviparous species. However, the growth rate of adult individuals did not follow this trend, presumably due to the weak association between growth and metabolism at this life stage. We further deduce that shark genome size modulation, as that of other fishes, is not subject to selective pressures stemming from water temperature or depth. This could be attributed to the significant impact temperature has on metabolic processes, potentially overriding any nucleotypic selective advantage. The prevalence of smaller genomes in marine-brackish and amphidromous species relative to purely marine ones contrasts the patterns found in ray-finned fishes and suggests that metabolic constraints related to osmoregulatory costs in fluctuating salinities may play a role. Finally, we found no support for a maladaptive nature of large DNA accumulations, as they did not affect the diversification of affected clades nor were influenced by neutral processes, like population size.

### Supplementary Information

Supplementary Information.

Supplementary Information.

## Data Availability

All trait data (described in “Data compilation” section of Methods, Supplementary Data S1–S4), phylogenies (from “The phylogeny” section, Supplementary Data S5, S6), and ChromEvol output files (from “Chromosome number evolution” section, Supplementary Data S7–S9) generated during this study, together with the R-script (Supplementary Code), are available in the figshare repository at: https://figshare.com/s/e8fb3790edb95f605576.
