# Supplementary material for: Shark genome size evolution and its relationship with cellular, life-history, ecological, and diversity traits
Source: Sci Rep. 2024 Apr 17;14:8909. doi: 10.1038/s41598-024-59202-4 (PMC11024215; doi:10.1038/s41598-024-59202-4)
Supplement: Supplementary file 1 — Supplementary Information. [file 41598_2024_59202_MOESM1_ESM.pdf]

Supplementary Information I for

Shark genome size evolution and its relationship with cellular, life-history, ecological, and diversity traits

Mario Torralba Sáez[1,2], Michael Hofreiter[3] & Nicolas Straube[4]

[1]Ichthyology section, Bavarian State Collection of Zoology (SNSB-ZSM), 81247 Munich, Germany
[2]Systematic Zoology, Department Biology II, Faculty of Biology, Ludwig Maximilian University of Munich (LMU), 82152 Munich, Germany
[3]Evolutionary Adaptive Genomics, Institute for Biochemistry and Biology, University of Potsdam, 14476 Potsdam, Germany
[4]Department of Natural History, University Museum of Bergen, University of Bergen (UiB), 5007 Bergen, Norway

This PDF file includes:

- Supplementary Methods
    - Definitions (and references) for the cytological, life-history, ecological, and diversity parameters used in comparative analysis.
    - Conventional simple regression analysis.
- Supplementary Tables S1 to S11.
- Supplementary Figures S1 to S10.
- References for Supplementary Information I.

Supplementary Methods

Definitions (and references) for the cytological, life-history, ecological, and diversity parameters used in comparative analysis

Cytological factors

Cell and nucleus sizes: measured (in μm^2) from erythrocyte dry smears as cell area (***C_a***) and nucleus area (***N_a***), respectively. Estimates were collected from the Cell Size Database[1] and the literature[2–4]. Given the flattened elliptical shape of mature erythrocytes in sharks (unlike in mammals, for example), these values were calculated from the long (*LD*) and short (*SD*) diameters of erythrocyte dry smears using the equation for elliptical area (*A*):

$$A = \pi \times \left(\frac{LD}{2}\right) \times \left(\frac{SD}{2}\right)$$

Morphological factors

-Body size, assessed by:

Total body length: estimates of minimum and maximum common total body length at maturity for males and females, and of maximum recorded total body length for each species—measured (in cm) from the nose to the end of the tail in a straight line—were extracted from the literature[5] and the online databases FishBase[6] and Shark-References[7]. For maximum length measurements, the upper caudal fin lobe was bent to the level of the caudal peduncle. The different species-specific estimates of length were used to conduct a Principal Component Analysis (PCA). The resulting coordinates of the first principal component, or PCA1 scores (***L_{PCA1}***), which summarized 96.36% of the length data variation observed, were then used in comparative analysis.

Maximum body weight (*Wmax*): maximum recorded body weight (in kg) collected from FishBase[6] and Shark-References[7].

-Body form: In a recent investigation on the diversity of body forms in extant shark species, Sternes and Shimada (2020)[8] suggested that the morphology of the precaudal portion of the body has the strongest influence on shark body form, followed by the shape of the caudal fin. As such, body form was assessed by:

Precaudal body shape (*PBS*): for this variable, the four classic body groups (body types 1 to 4) proposed by Thomson and Simanek (1977)[9] were preferred over the two basic body plans (“deep-bodied” and “shallow-bodied”) identified by Sternes and Shimada (2020)[8] across the precaudal portion of the shark’s body in its lateral view. This decision was based on the over-generalization of body form and associated swimming mechanics acknowledged by the latter study. Definitions:

- Body type 1: includes sharks characterized by a conical head, a large and deep body, large pectoral fins, reduced pelvic, second dorsal, and anal fins; and a narrow caudal peduncle with lateral keels. These are typically fast-swimming pelagic sharks such as *Carcharodon, Isurus,* and *Lamna*.
- Body type 2: compared to the previous group, these sharks have more blunt heads, more ventrally flattened heads and bodies (hence, less deep), large pectoral fins, moderately sized pelvic, second dorsal, and anal fins; and lacking keels. This body form, generally typified by the Carcharhinidae, corresponds to more generalized, continental swimmers.
- Body type 3: sharks with very large heads and blunt snouts, more anterior pelvic fins, and more posterior first dorsal fins. These include slow-swimming epibenthic, benthic, and demersal sharks, such as the Carcharhiniformes Scyliorhinidae and Triakidae, Hexanchiformes, and most Orectolobiformes.

- Body type 4: these sharks lack an anal fin and often have higher pectoral fin insertions (more in line with the external branchial openings). This group is exclusively represented by squaliform species (except for Hexanchiformes), which includes many deep-sea and some continental species (like *Squalus*) with generally very slow cruising speeds.
- Body type 5: this additional category was included in the present study to characterize acute dorsoventrally flattened species, such as the epibenthic Squatiniformes, found normally resting on the bottom.

Caudal fin aspect ratio (*CFAR*): calculated as caudal fin height squared divided by its surface area. This number is treated as an indicator of swimming mode—high values are linked to fast-swimmers[10,11]—and therefore, as a correlate of the average level of organismal activity. *CFAR* data was mostly extracted from FishBase[6], but also from the literature[10,12]. When there was more than one value for a single species, estimates based on pictures (if available) were prioritized over the ones estimated from illustrations (i.e., poor-quality values). Analysis was run over the entire data compiled, but also discarding poor-quality values.

Metabolic and physiological factors

Standard metabolic rate (*SMR*): measured as mass-specific oxygen consumption rate at rest during fasting (VO_2, in mg O_2 kg^{-1} h^{-1}). Estimates were mainly retrieved from Carrier et al. (2012, Table 7.1; references therein)[13], but also from more recent sources concerning some species-specific estimates[14–17]. Note that *SMR* in ram ventilator species is estimated by extrapolation to "zero velocity": the *y*-axis intercept in the power performance curve (i.e., the relationship between oxygen consumption and swimming speed). Given the major influence of temperature on metabolic rate in ectothermic elasmobranchs[13], VO_2 values were corrected to 20 ºC using a fish-specific temperature coefficient (Q_{10}) of 1.65[18] in the temperature coefficient equation. Normalization to a common temperature was then accomplished by isolating R_2 (the expected VO_2 at 20 ºC) in the temperature coefficient equation (see below, where R_1 is the standard metabolic rate from the original study, T_1 is the temperature at which R_1 was measured, and T_2 is 20 ºC):

$$Q_{10}=\left(\frac{R_2}{R_1}\right)^{\left(\frac{10}{T_2-T_1}\right)}$$

Cruising speed: speed of locomotion in sharks has been found to be fairly accurately predicted as a function of body length (fork length, FL) and tail shape (*TS*, tail types 1 to 4; following Thomson and Simanek (1977)[9])[19]. The scattered availability of shark FL estimates prevented the calculation of a large enough sample of cruising speed estimates within our dataset. However, given the strong correlation between FL and total body length in sharks ($r > 0.87$)[20], a regression model including "Total body length (L_{PCA1}) + *TS*" as predictors was used instead for estimating the relationship between important morphological factors related to cruising speed and genome size. This approach also allowed us to disentangle the effects of body size allometric scaling from the morpho-ecological implications regarding tail morphology involved in the shark's speed of locomotion. Definitions:
- Tail type 1: including externally symmetrical tails with a very high-aspect ratio (half-moon shape).
- Tail type 2: tails characterized by significantly lower heterocercal angles than tail type 1.
- Tail type 3: very low (almost straight) tails with a small to absent ventral hypochordal lobe, a large longitudinal hypochordal lobe, and a large subterminal lobe.
- Tail type 4: tails characterized by the presence of a large epicaudal lobe.

For a given body size, sharks with tail type 2 have the highest cruising speeds, followed by those with tail type 1, subsequently followed by sharks with tail type 3, and finally by sharks with tail type 4, which are characterized by the lowest cruising speeds among all[19].

Developmental and demographic factors

Developmental and demographic parameters used in other studies of genome size diversity (such as developmental times at different ontogenic stages or population doubling times) are very scarce for sharks. To compensate for it, we used:

Growth completion rate (*k*): this parameter, derived from the von Bertalanffy growth function[21], indicates the rate (in $years^{-1}$) at which the asymptotic length, the ultimate length reached if fish were to grow indefinitely, is approached. When several values were available for a single species, the geometric mean was used (see sources below).

Age: as for total body length, a PCA was run over the estimates (in years) of age at maturity for males and females, and of maximum recorded lifespan for each species. The first principal component coordinates, or PCA1 scores (T_{PCA1}), which comprised 98.36% of the total age data variation, were then used in comparative analysis.

Overall, age- and growth-related data were obtained from the literature[5,13,22–32] and the online databases AnAge[33], FishBase[6], and IUCN Red List[34]. References therein were carefully checked, given the recurrent spread of typos and unreliable estimates regarding these parameters (at least for sharks). Estimate selection is explained in detail in the comments attached within Supplementary Data S3 online. For species for which there are no developmental studies available to date, values were estimated using the FishBase Life-history tool (i.e., poor-quality values) when possible, which makes use of the von Bertalanffy and derived growth functions (see https://www.fishbase.se/manual/key%20facts.htm). The default life-history values used in the growth equations by the aforementioned tool were substituted by the ones collected in the present study where allowed by the interface. Analysis was performed over the entire dataset, but also discarding these poor-quality estimates.

Maximum intrinsic rate of population increase (r_{max}): rate (in $years^{-1}$) at which population size increases in the absence of density-dependent regulation (a standard measurement of population productivity). Data were collected exclusively from Pardo et al. (2016)[35], since only this study considers shark's survival to sexual maturity, thereby enhancing the credibility of the r_{max} estimates.

Reproductive factors

Reproduction mode: three categories were assessed corresponding to the diverse reproductive strategies adopted by sharks, including "oviparous" (species in which females deposit eggs), "aplacental viviparous", previously known as ovoviviparous (including live-bearing species in which the embryos are nourished by the yolk in their yolk sac (*lecithotrophy*), by unfertilized eggs (*oophagy*), or by other embryos (*adelphophagy*)), and "placental viviparous" (live-bearing in which the embryos feed from placental connections (*placentotrophy*) as the yolk sac develops into a placenta inside the maternal uterus).

Litter size (L_s): measured as average litter size per gestation period. When a range of values was available, the midpoint was taken as the average. For both reproductive parameters, data were collected from the literature[5,13], with small details appended from FishBase[6].

Ecological factors

Preferred water temperature: mean preferred water temperature (in ºC), calculated from large occurrence datasets compiled within the AquaMaps tool, available from FishBase[6].

Depth: assessed (in m) by average depth and depth range:

Average depth: midpoint between the depth limits at which the species is commonly found.

Depth range: difference between the maximum and the minimum depths at which the species has been reported. For both cases, depth data was collected from the literature[5], with some details appended from FishBase[6], Shark-References[7], and the IUCN Red List[34].

Climate: categorized as “tropical”, “subtropical”, “temperate”, “boreal”, and “worldwide” (defined as oceanodromous migratory species found circumglobally) according to the species maximum latitude range distribution.

Occurrence: species living and feeding areas across the water column, including the three categories “pelagic” (free swimming, living and feeding in the open sea, in association with the surface or middle depths of a body of water), “benthopelagic” (living near the bottom as well as in mid-waters or near the surface, feeding on benthic as well as free swimming organisms. Pertaining to forms that hover or swim mainly near or just above the sea floor but do not rest on the substrate), and “epibenthic” (sinking to or lying on the sea floor, living on the bottom and feeding on benthic organisms). Additional categories based on water-depth (e.g., bathypelagic, bathydemersal, etc.) were not included. Instead, (ln-transformed) average depth was integrated as a covariate in the models designed to analyse genome size differences across occurrence categories. Depth corrections were also applied to models analysing the effects of climate (above, to only account for clinal differences in temperature) and salinity (below, given the conspicuous differences in water-depth existing between marine and non-marine environments).

Habitat: categorized as “reef-associated” (living and/or feeding on or near coral reefs, between 0 and 50 m deep), “coastal” (living in waters within the 200 m depth contour of the continental shelf, including shelf or neritic (inshore) waters, protected bays, and estuaries), “oceanic” (pertaining to the open ocean (offshore) beyond the continental and insular shelves, in waters extending over continental slopes, ocean floors, sea mounts, and abyssal trenches at depths above 200 m), and “deep-water” (living in open (offshore) waters or fjords at depths below 200 m).

Salinity: preferred water salinity, defined by the categories “marine” (living in purely marine waters), “marine-brackish” (living or frequently venturing waters that are more saline than freshwater but less saline than true marine environments, like estuaries, lagoons, or enclosed bays), and “amphidromous” (for those species capable of entering rivers).

The categories of the four last qualitative variables were assigned based on the species information given by the literature[5], FishBase[6], and Shark-References[7], in agreement with the definitions provided here.

Diversity factors

Taxonomic diversity: a total of five taxonomic diversity parameters were taken into account, including the number of species per family and per order, the number of genera per family and per order, and the number of families per order, following the most updated taxonomy given in Shark-References[7].

Genetic diversity: assessed by expected heterozygosity ($\boldsymbol{H_e}$)[36], calculated most commonly from CR mtDNA markers but also from other mitochondrial markers (such as ND2, ND4, CytB, and COI) as well as from microsatellites, AFLPs, RFLPs, and SNPs. When several estimates were available for a single species, obtained from a literature review[37], the average value was used.

Further information regarding the species-specific parameter estimates used and associated original references for each variable can be found in Supplementary Data S3 and S4 online.

Conventional simple regression analysis

All the relationships between genome size and the aforementioned biological parameters (as explained in Section 3.3.1 of Methods) were also tested via conventional (non-phylogenetically corrected) Ordinary Least Squares (OLS) regression analysis, using the R package *car* v. 3.1.2[38], for comparison with previous reports on different taxa (summary statistics are given in Supplementary Table S5).

Given the nested structure of the taxonomic diversity data (e.g., species belonging to the same "family" will account for the same "number of species per family"; Durbin–Watson test: $DW < 0.75$, $p < 0.001$*** for the OLS regression residuals of all taxonomic diversity parameters against genome size), we applied non-phylogenetically corrected (1) mixed-effects linear regressions, via the *nlme* R package v. 3.1.164[39], with taxonomy as the random effect (in the example, "family"); and (2) OLS regressions of averaged values at the corresponding taxonomic scale [*car* package], following Olmo (2006)[40] in his respective study across vertebrates (in the example, family-level averaged C-values regressed against the respective "number of species per family"). These steps were not required under a PGLS framework, as the nested taxonomic hierarchy is based on common ancestry, and thus residual autocorrelations were automatically *dissolved* (i.e., not detected) during phylogenetic corrections.

For OLS regression analysis, outliers (see Section 3.3 of Methods) included additionally data points with a Cook's distance > 0.5, which were detected through the 'influencePlot' function implemented in the *car* package (note that this analysis is still not developed for PGLS regression analysis).

Supplementary Table S1. Summary of haploid genome size and karyotype parameters across shark species. Four chimaera species and the batoid *Hypanus americanus*, used as an outgroup in ancestral reconstruction analyses, are also included. For genome size, average values for species with more than one estimate (only 18) were calculated, with coefficients of variation (CV) for the multiple entries shown in parenthesis (note that no CV exceeded 25—with 30 generally accepted as a value indicating considerable variation). Regarding karyotype composition, two-armed chromosomes were represented by metacentric (M) and submetacentric (SM) chromosome morphotypes, while subtelocentric (ST), acrocentric (A), and telocentric (T) chromosomes and microchromosomes (MC) were classified as one-armed (classification of chromosomes followed Levan et al. 1964[41]). When several karyotype count data were available for a single species, karyotype composition was based on the source from which the most recent/reliable values for chromosome number (n) and fundamental number (FN) of chromosome arms (i.e., underlined numbers in the second and third columns, respectively) were obtained. A reference list for all the estimates used can be found in Supplementary Data S1 and S3 online. Note that numerous alternative FN values proposed mainly by Schwartz and Maddock (2002)[42] were not considered for analysis (nor included in the present table) given the constant discrepancies with the numbers provided by other authors. These differences were potentially caused by different criteria for karyotype classification (i.e., one-armed *vs.* two-armed chromosome counting).

Lineage / Order / Species	Genome size (pg/n) ± SE (CV)	Chromosome number (n)	Fundamental number (FN)	Karyotype Composition: Two-armed chromosomes (M-SM)	Karyotype Composition: One-armed chromosomes (ST-A-T-MC)
**Holocephali**					
**Chimaeriformes**					
Callorhinchus milii[a]	1.94	-	-	-	-
Rhinochimaera pacifica[a]	1.58	-	-	-	-
Chimaera monstrosa[a]	-	43	43	0	43
Hydrolagus colliei[a]	1.56 ± 0.05 (4.09)	29	29	0	29
**Batoidea**					
**Myliobatiformes**					
Hypanus americanus[a]	4.60 ± 0.95 (29.21)	39	43.50	34.50	4.50
**Selachimorphii (or Selachii)**					
**Heterodontiformes**					
Heterodontus francisci	7.60 ± 0.59 (13.43)	51	64	13	38
Heterodontus japonicus	14.80	51	56	5	46
**Orectolobiformes**					
Orectolobus ornatus	5.05	-	-	-	-
Hemiscyllium ocellatum	5.51	54	73	19	35
Chiloscyllium plagiosum	4.96	53 / 51	78 / 83	25	28
Chiloscyllium punctatum	4.82 ± 0.10 (4.16)	53 / 52	79	26	27
Ginglymostoma cirratum	3.90 ± 0.10 (3.63)	-	-	-	-
Rhincodon typus	3.83	51	59	8	43
Nebrius ferrugineus	4.25	-	-	-	-
Stegostoma tigrinum	3.79	51	61	10	41
**Lamniformes**					
Megachasma pelagios	5.81	-	-	-	-
Carcharias taurus	6.55	42	66	24	18
Cetorhinus maximus	4.31[†]				
Carcharodon carcharias	6.45	41	65	24	17

Lineage<br>Order<br>Species	Genome size (pg/n) ± SE (CV)	Chromosome number (n)	Fundamental number (FN)	Karyotype Composition	
				Two-armed chromosomes (M-SM)	One-armed chromosomes (ST-A-T-MC)
Isurus oxyrinchus	5.86 ± 0.84 (20.27)	-	-	-	-
**Carcharhiniformes**					
Scyliorhinus canicula	6.20 ± 0.65 (18.17)	31 / 40**	52	21	10
Scyliorhinus stellaris	6.15	36	61	25	11
Scyliorhinus torazame	6.72 ± 0.12 (2.42)	32	45	13	19
Cephaloscyllium umbratile	7.35	32	49	17	15
Cephaloscyllium ventriosum	7.88 ± 0.63 (13.78)	32	55	23	9
Atelomycterus marmoratus	4.33[†]	38	64	26	12
Galeus eastmani	5.50	-	-	-	-
Galeus melastomus	6.15	-	-	-	-
Galeus nipponensis	5.55	-	-	-	-
Triakis scyllium	4.90	36	54	18	18
Triakis semifasciata	4.80	36 / 35*	62 / 61*	26	10
Galeorhinus galeus	8.65	-	-	-	-
Mustelus asterias	4.30	-	-	-	-
Mustelus manazo	4.65	34 / 36	56 / 42	22	12
Mustelus californicus	6.40	-	-	-	-
Mustelus canis	4.40 ± 0.20 (6.43)	40	62	22	18
Mustelus norrisi	4.50	-	-	-	-
Galeocerdo cuvier	4.86 ± 0.61 (25.25)	43	63 / 62*	20	23
Sphyrna lewini	3.58 ± 0.30 (17.02)	43 / 41 / 39*	53 / 56 / 48*	10	33
Sphyrna tiburo	3.90	-	-	-	-
Rhizoprionodon porosus	3.90	-	-	-	-
Rhizoprionodon terraenovae	3.60	45 / 40*	62	17	28
Negaprion brevirostris	3.70	-	-	-	-
Carcharhinus limbatus	3.69 ± 0.23 (12.35)	43 / 40*	60 / 55*	17	26
Carcharhinus melanopterus	3.00	-	-	-	-
Carcharhinus amblyrhynchos	3.42	-	-	-	-
Prionace glauca[b]	4.30	43 / 39	58 / 44	15	28
Carcharhinus brachyurus	2.86	-	-	-	-
Carcharhinus plumbeus	3.21 ± 0.23 (9.93)	37 / 38**	46	9	28
Triaenodon obesus	2.88	-	-	-	-
Carcharhinus acronotus	3.52 ± 0.07 (3.50)	43 / 42*	61 / 58*	18	25
Carcharhinus perezii	5.85	-	-	-	-
Carcharhinus longimanus	3.34	-	-	-	-
Carcharhinus galapagensis	4.25	-	-	-	-
Carcharhinus obscurus	2.87 ± 0.14 (6.66)	39	49	10	29

Lineage<br>Order<br>Species	Genome size (pg/n) ± SE (CV)	Chromosome number (n)	Fundamental number (FN)	Karyotype Composition	
				Two-armed chromosomes (M-SM)	One-armed chromosomes (ST-A-T-MC)
**Hexanchiformes**					
Chlamydoselachus anguineus	4.59	50	56	6	44
Notorynchus cepedianus	4.40	52	54 / 58** / 55**	2	50
Heptranchias perlo	-	36	39	3	33
Hexanchus griseus	5.35	-	-	-	-
**Squatiniformes**					
Squatina squatina	9.80	-	-	-	-
Squatina australis	16.41	-	-	-	-
Squatina californica	9.30	44	57	13	31
**Squaliformes**					
Centrophorus squamosus	6.55	-	-	-	-
Deania calcea	7.19 ± 0.09 (1.67)				
Somniosus microcephalus	10.62	-	-	-	-
Centroselachus crepidater	12.29 ± 0.71 (8.17)	-	-	-	-
Centroscymnus coelolepis	10.99 ± 0.86 (11.07)	-	-	-	-
Oxynotus bruniensis	12.49	-	-	-	-
Oxynotus centrina	17.05	31	59	28	3
Scymnodon ringens	8.20	-	-	-	-
Centroscymnus owstonii	10.17	-	-	-	-
Scymnodon macracanthus	9.76	-	-	-	-
Dalatias licha	9.10	-	-	-	-
Squalus acanthias	6.58 ± 0.28 (9.60)	32 / 30 / 29 / 31 / 39**	48 / 60 / 57 / - / 58**	16	16
Squalus suckleyi	-	31	41	10	21
Etmopterus pusillus	8.11	43	43	0	43
Etmopterus brachyurus	11.89	-	-	-	-
Etmopterus molleri	13.34	-	-	-	-
Etmopterus granulosus	12.71	-	-	-8	-
Etmopterus spinax	16.15	43 / 31**	43 / 36**	0	43

[a] Species included as an outgroup in ancestral state reconstruction analyses.
[b] Species taxonomic note from Shark-References[7]: da Silva Rodrigues-Filho et al. (2023)[43] conclude that *Prionace glauca* is reclassified and recognized as *Carcharhinus glaucus*, even though the topic is still being discussed.
[§] Genome size value appearing in the Squalomix data repository[44], but not referenced or absent in the original reference specified and not found elsewhere (not considered for analysis).
* Karyotype value from Schwartz and Maddock (1986)[45] later changed by the same authors[42] to the value without "*" for the same species (not considered for analysis).
** Invalid karyotype value due to typos from review sources or unreliable chromosome counting from poor-quality photographs (not considered for analysis).

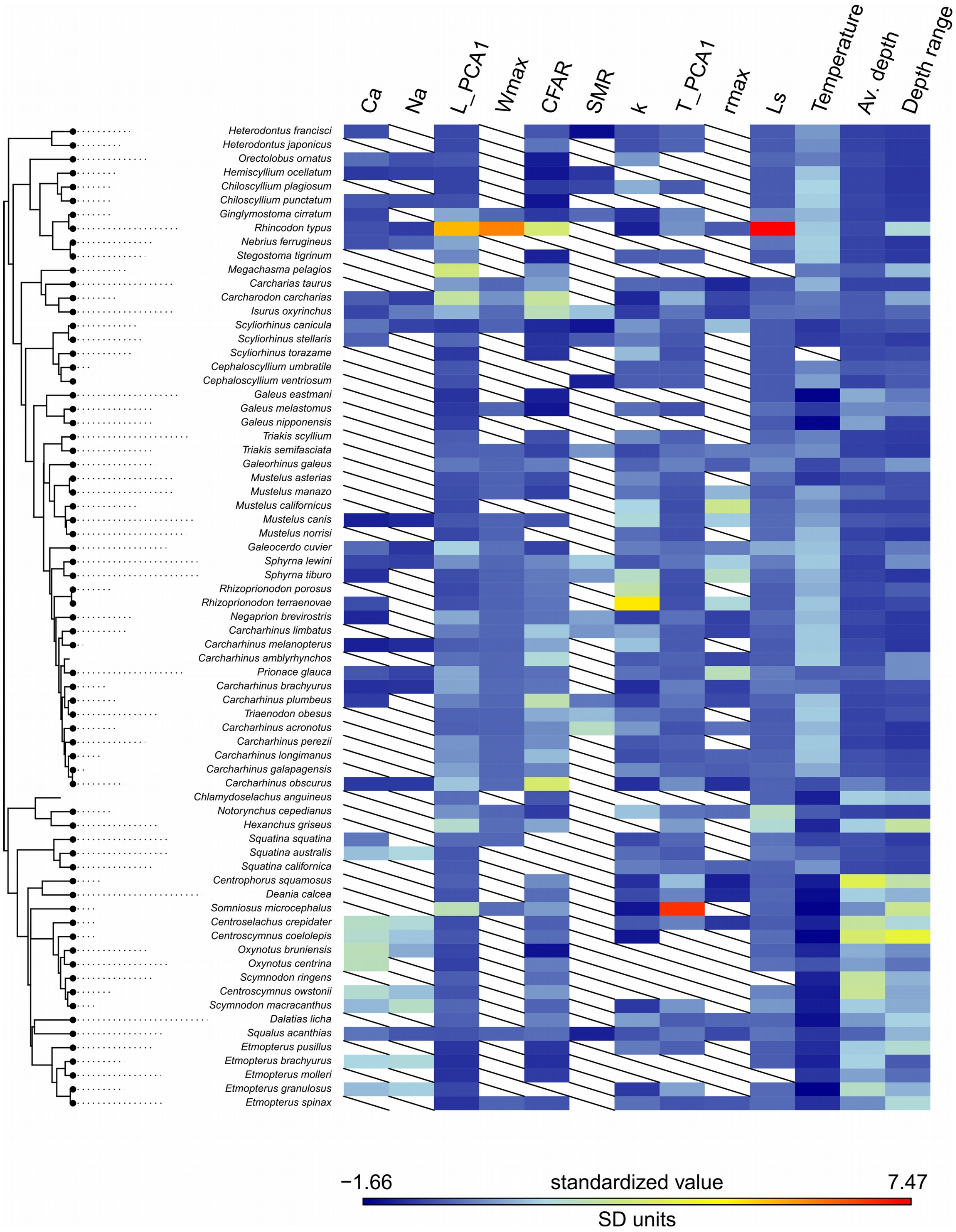

Supplementary Figure S1. Multivariate phylogenetic heatmap of quantitative biological data. The heatmap visualizes species data distribution (with unknown values as crossed squares) for the quantitative cytological, life-history, and ecological parameters used in comparative analysis. Prior to colour representation (bottom inset), parameter estimates were standardized to have the same mean and variance equal to 1 (note that outliers detected in regression analysis are included).

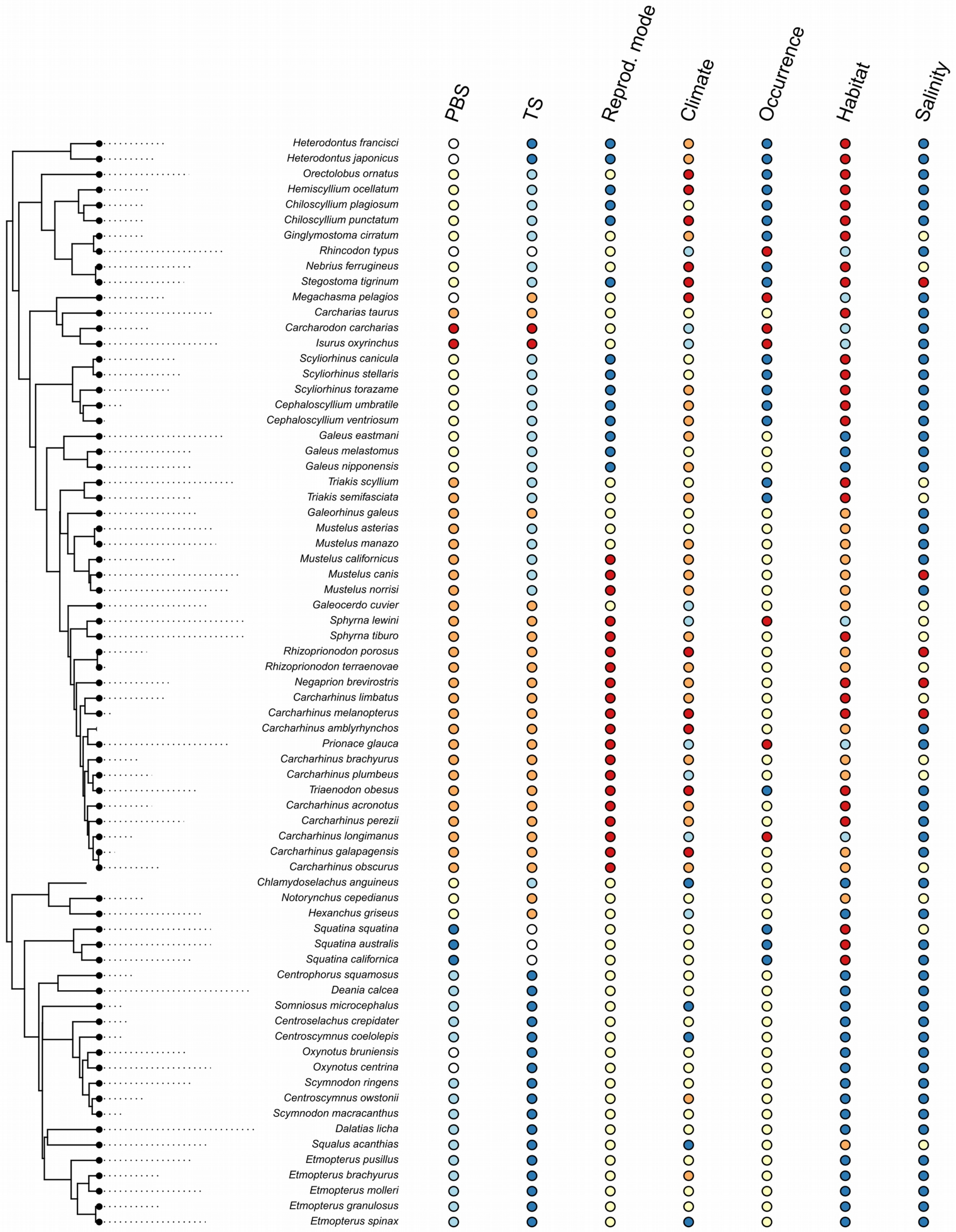

Supplementary Figure S2. Multivariate phylogenetic dotplot of qualitative biological data. The dotplot visualizes category assignment (with unknown states as empty circles) for the qualitative life-history and ecological parameters used in comparative analysis. The colour scheme used corresponds to that of Supplementary Figures S8 and S9, where cold colours represent more K-selected traits and ecologies and warm colours represent more r-selected ones (note that outliers detected in regression analysis are included).

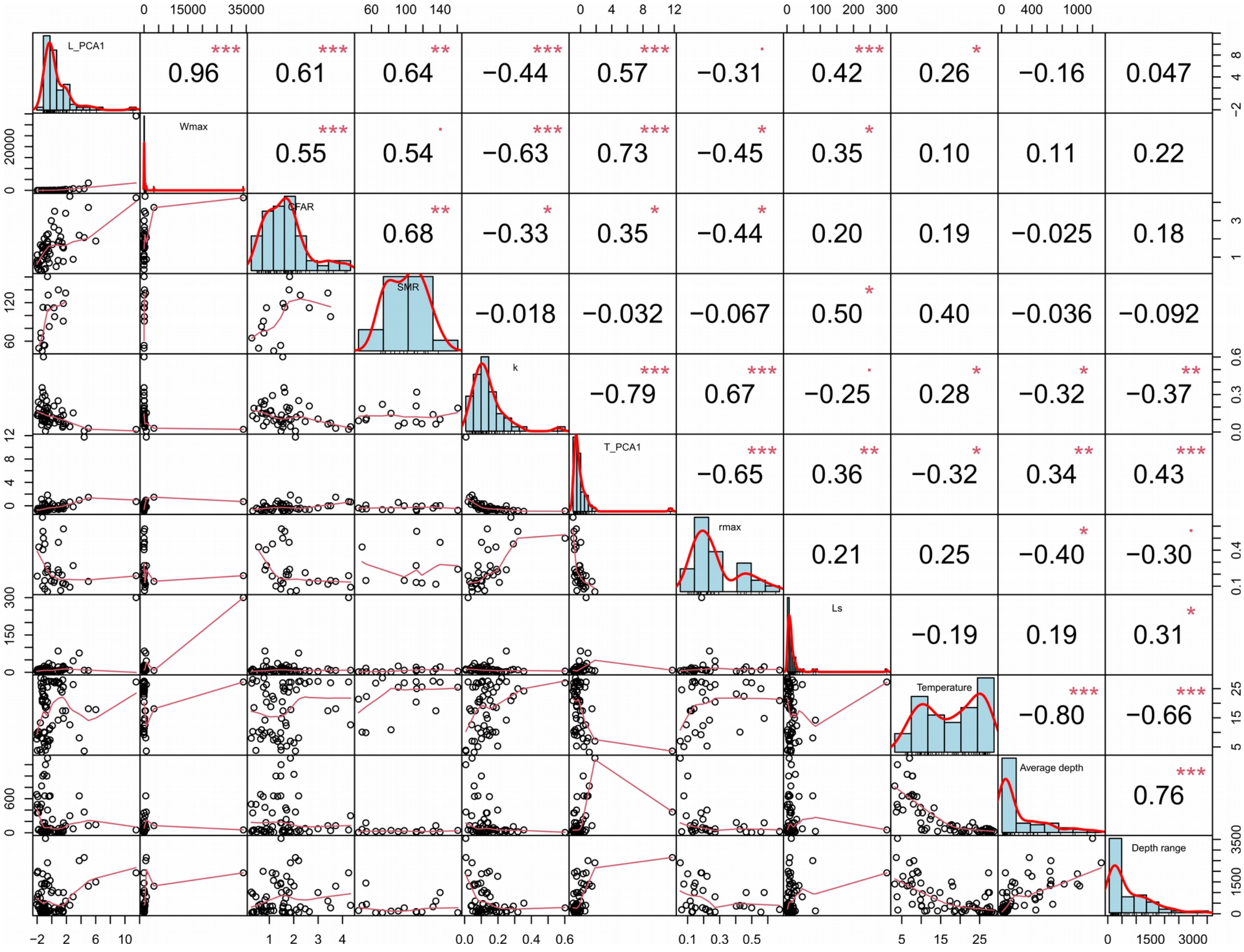

Supplementary Figure S3. Correlation structure of quantitative biological data. Spearman's rank-order correlation matrix showing pairwise relationships between the quantitative life-history and ecological parameters used in comparative analysis. Top-right: the (absolute) values of each correlation test and associated *p*-values (**p* ≤ 0.05; ***p* ≤ 0.01; ****p* ≤ 0.001). Bottom-left: the bivariate scatterplots, with a fitted line in red (note that parameter estimates are un-transformed and outliers detected in regression analysis against genome size included—to which Spearman’s correlation analysis is not very sensitive). This figure required the additional R package *PerformanceAnalytics* v. 2.0.4[46].

Supplementary Table S2. Model-fitting results for analyses of shark genome size evolution. Summary statistics are provided for analysis including (*With outg.*) and excluding (*Without outg.*) an outgroup in the 'Maximum Clade Credibility' (MCC) tree run under the 'Brownian Motion' (BM), the 'Ornstein-Uhlenbeck' (OU), and the 'Early-Burst' (EB) models of evolution (best-fitting models in bold). Logarithmic likelihood (Log-Lik) and AIC scores (AICc and AICw) for each of the models tested are given, along with the parameter estimates θ (phylogenetic trait mean), σ^2 (evolutionary rate), α (elastic band parameter, in OU), and "a" (evolutionary rate change parameter, in EB). Robustness of results to phylogenetic uncertainty is shown in Supplementary Figure S5.

Tree	Model	Log-Lik	AICc	ΔAICc	AICw	Parameter estimates
	BM	-9.056	24.450	3.853	0.122	$\theta = 1.382$; $\sigma^2 = 0.0005$
With outg.	OU	-9.056	26.683	6.086	0.040	$\theta = 1.382$; $\sigma^2 = 0.0005$; $\alpha = 0$
	**EB**	**-6.013**	**20.597**	**0.000**	**0.838**	**$\theta = 1.264$; $\sigma^2 = 0.0036$; a = -0.0076**
	**BM**	**-3.132**	**12.621**	**0.000**	**0.497**	**$\theta = 1.913$; $\sigma^2 = 0.0004$**
Without outg.	OU	-3.132	14.869	2.248	0.162	$\theta = 1.913$; $\sigma^2 = 0.0004$; $\alpha = 0$
	EB	-2.383	13.372	0.750	0.342	$\theta = 1.913$; $\sigma^2 = 0.0010$; a = -0.0059

Supplementary Table S3. Pagel's tree transformation parameter results. Summary statistics, including logarithmic likelihood (Log-Lik), AICc score, and the corresponding Pagel's parameter estimate, are shown for analysis run under the MCC tree including (*With outg.*) and excluding (*Without outg.*) an outgroup. Each model providing the maximum likelihood value of its corresponding Pagel's parameter *estimate* was compared, via likelihood-ratio test (LRT), against null models (H_o) where *estimate* = 0 (for λ and κ) and H_o where *estimate* = 1 (for all parameters). Superscripts following each Pagel's parameter *estimate* indicate the *p*-values obtained from these LRT tests (first position: against H_o *estimate* = 0; second position: against H_o *estimate* = 1; significant results in bold: $*p \leq 0.05$; $**p \leq 0.01$; $***p \leq 0.001$), followed (in parenthesis) by the percentage of trees from the 100 alternative phylogenies rendering a *p*-value falling on the same side of the significance threshold ($\alpha = 0.05$) as inferred for the MCC tree. Robustness of the actual Pagel's parameter estimates to phylogenetic uncertainty is shown in Supplementary Figure S5.

Parameter	Tree	Log-Lik	AICc	Estimate$^{\textit{p}\text{-value } \textit{vs.} \text{ Ho } \textit{est.} = 0; \textit{p}\text{-value } \textit{vs.} \text{ Ho } \textit{est.} = 1}$
Lambda (λ)	*With outg.*	-8.931	26.434	$0.981^{\mathbf{8.63e\text{-}23^{***}\ (100\%)};\ 0.6172\ (100\%)}$
	Without outg.	-2.993	14.592	$0.967^{\mathbf{1.16e\text{-}20^{***}\ (100\%)};\ 0.5988\ (100\%)}$
Delta (δ)	*With outg.*	-6.253	21.078	$0.206^{\mathbf{0.0179^{*}\ (62\%)}}$
	Without outg.	-2.691	13.987	$0.606^{0.3476\ (99\%)}$
Kappa (κ)	*With outg.*	-9.024	26.619	$0.909^{\mathbf{0.0248^{*}\ (73\%)};\ 0.7994\ (99\%)}$
	Without outg.	-2.604	13.814	$0.586^{0.1766\ (95\%);\ 0.3044\ (100\%)}$

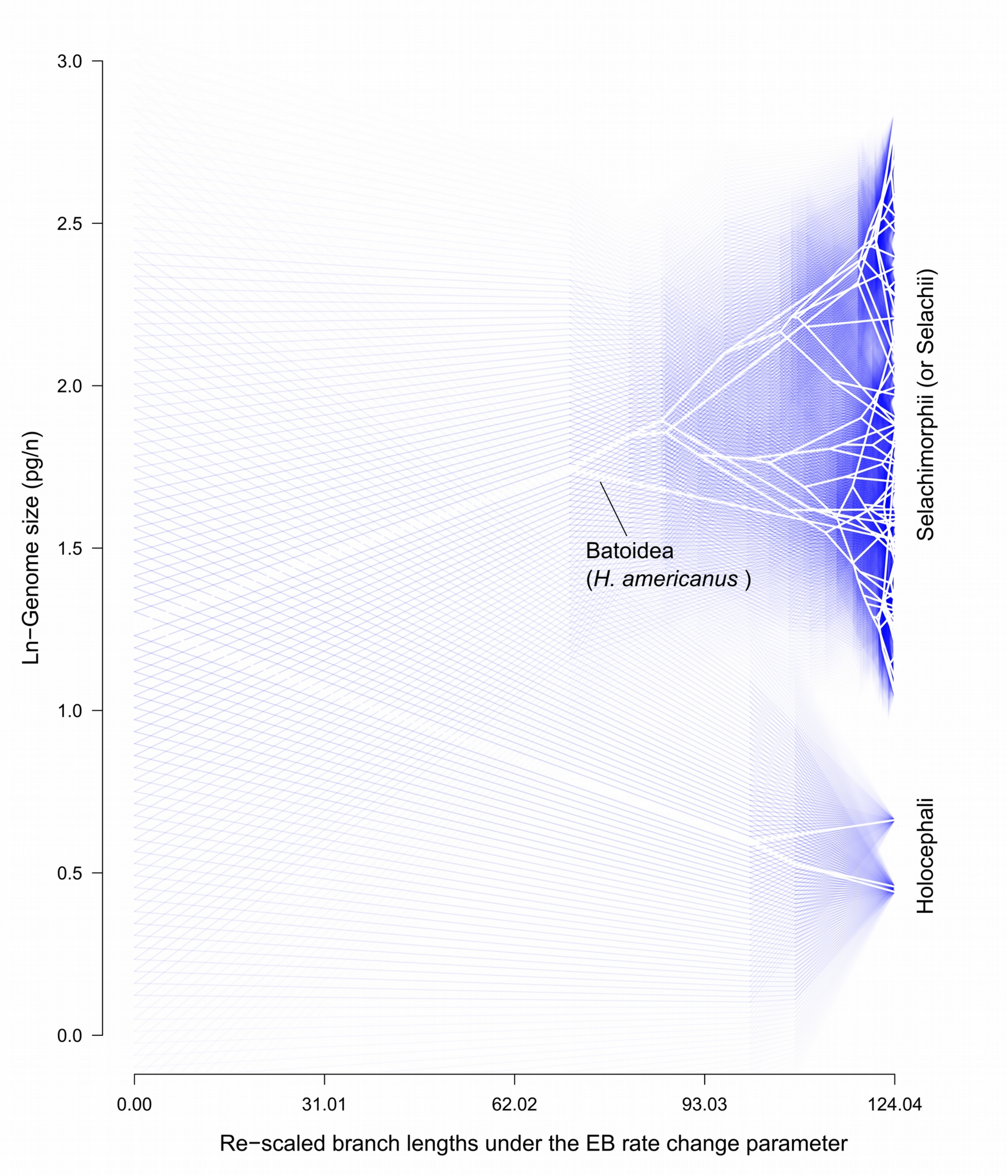

Supplementary Figure S4. Projection of the phylogeny onto genome size phenotypic space. Taitgram (or phenogram) depicting 95% uncertainty in ancestral (ln-transformed) genome size reconstruction under the 'Early-Burst' (EB) model using transparent probability density. The phylogeny corresponds to the MCC tree hypothesis and includes the outgroup species. Branch lengths are re-scaled under the EB evolutionary rate change parameter ("a" in Supplementary Table S2), revealing that high rates of change concentrated early in the tree (with a significant genome expansion and contraction in the lineages preceding the diversification of extant elasmobranchs (Selachimorphii and Batoidea) and chimaeras (Holocephali), respectively).

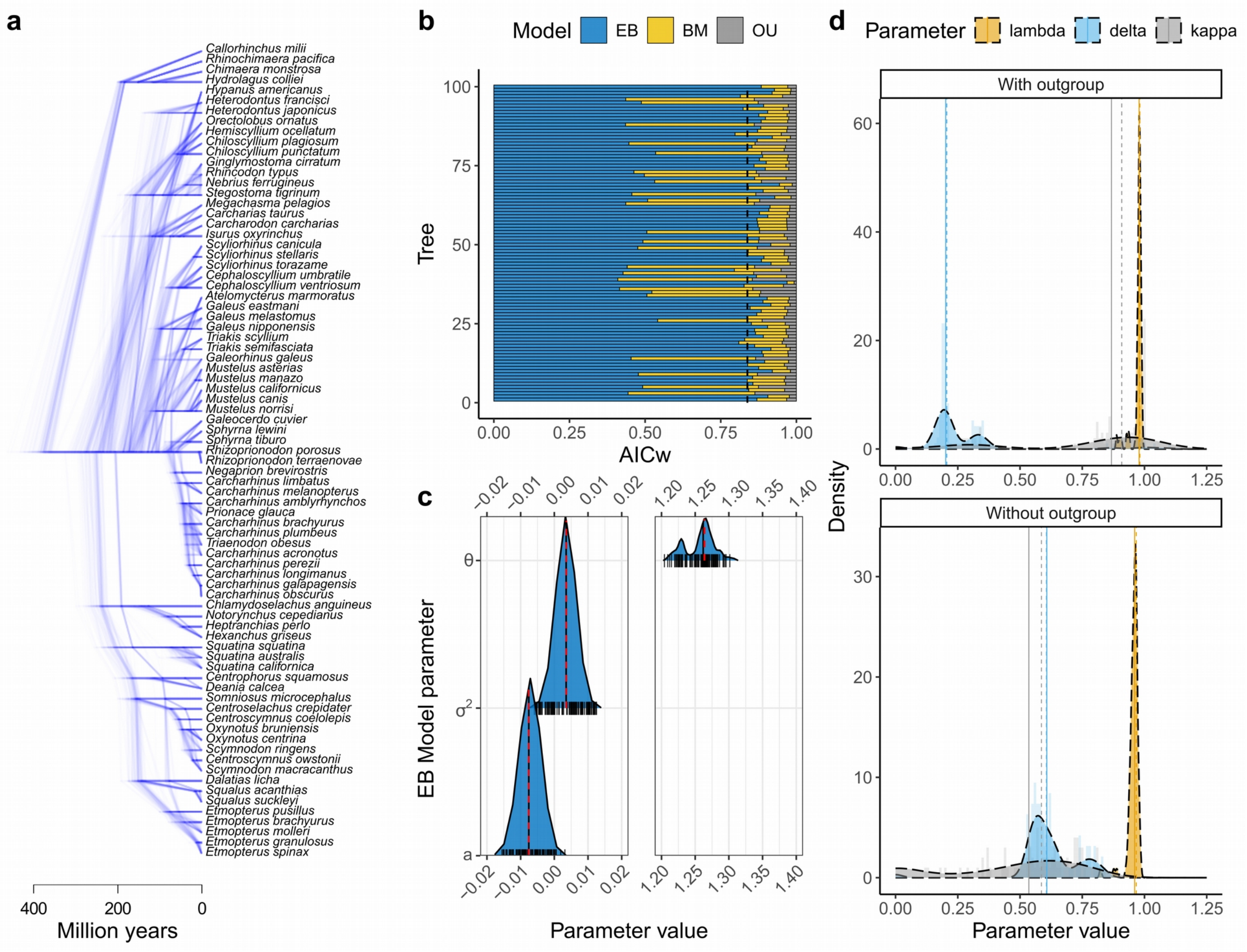

Supplementary Figure S5. Robustness of results to phylogenetic uncertainty in analyses characterizing the evolution of shark genome size. a, Posterior sample depicting variation in tree topology and branch lengths across the 100 alternative phylogenetic hypotheses considered. **b**, Stacked barplot showing the relative AICw value for each of the three models of genome size evolution (EB: 'Early-Burst', OU: 'Ornstein-Uhlenbeck', BM: 'Brownian Motion') tested over the 100 trees. The EB model, which had the highest AICw under the MCC tree (dashed line; Supplementary Table S2), was also the best-fitting model in 97% of the trees, confirming that this model better explains the evolutionary history of genome size evolution in sharks. However, this was true when an outgroup was included in the analysis, as the BM model performed better (i.e., had the highest AICw value) in analyses excluding the outgroup species both for the MCC tree (although with an AICc improvement score lower than 2; Supplementary Table S2) and for 89% of the alternative phylogenies (not visualized). The comparison of the rates of genome size evolution between shark superorders was also consistent across the alternative phylogenies (Mann–Whitney U test: W = 215–297, p < 0.01**, and n = 69 for all trees; not visualized). **c**, Density plot summarizing variation in the EB model parameters θ (phylogenetic trait mean), σ^2 (evolutionary rate), and "a" (evolutionary rate change parameter) over the 100 alternative trees. **d**, Density plot depicting variation in Pagel's parameter estimates over the 100 trees in analysis including (top) and excluding (bottom) an outgroup. For **c** and **d**, solid lines indicate median values across the different alternative trees, while dashed lines represent the corresponding estimates as inferred for the MCC tree. Panels **b** and **c** required the additional R packages *ggsci* v. 3.0.0[47], *ggridges* v. 0.5.6[48], and *ggbreak* v. 0.1.2[49].

Supplementary Table S4. Model-fitting results for the eight analyses of shark chromosome number (n) evolution carried out in ChromEvol. Logarithmic likelihood (Log-Lik) and AIC scores, together with the rate parameter estimates and total frequency of the four possible mechanisms of chromosome number (n) change with expectation > 0.5 are given for each of the models tested (best-fitting model in bold). Rate parameters, λ: single chromosome gain constant rate; λ_1: single chromosome gain rate linearly dependent on the current chromosome number; δ: single chromosome loss constant rate; δ_1: single chromosome loss rate linearly dependent on the current chromosome number; ρ: duplication (i.e., polyploidization) constant rate; μ: demi-duplication constant rate. Event types, Gains: nº of single chromosome gains; Losses: nº of single chromosome losses; Dupl.: nº of duplication (i.e., polyploidization) events; Demi.: nº of demi-duplication events.

				Rate parameters						Nº of events inferred			
**Model**	**Log-Lik**	**AIC**	**ΔAIC**	**λ**	**λ_1**	**δ**	**δ_1**	**ρ**	**μ**	**Gains**	**Losses**	**Dupl.**	**Demi.**
CONST_RATE	-120.41	246.80	2.60	42.50	-	23.48	-	~0.00	-	978.12	540.83	~0.00	0.00
CONST_RATE_DEMI	-120.38	246.80	2.60	37.21	-	28.02	-	~0.00	= ρ	855.91	644.66	~0.00	0.00
CONST_RATE_DEMI_EST	-120.36	248.70	4.50	34.36	-	31.41	-	~0.00	~0.00	789.55	721.97	~0.00	0.00
**CONST_RATE_NO_DUPL**	**-120.08**	**244.20**	**0.00**	**23.20**	**-**	**42.10**	**-**	**-**	**-**	**533.56**	**964.43**	**~0.00**	**0.00**
LINEAR_RATE	-120.33	250.70	6.50	3.41	0.50	21.31	0.50	~0.00	-	584.20	996.41	~0.00	0.00
LINEAR_RATE_DEMI	-120.33	250.70	6.50	3.41	0.50	21.31	0.50	~0.00	= ρ	583.65	995.90	~0.00	0.00
LINEAR_RATE_DEMI_EST	-121.47	254.90	10.70	28.54	-0.06	0.84	0.08	~0.00	0.46	608.64	78.37	0.15	9.79
LINEAR_RATE_NO_DUPL	-120.33	248.70	4.50	3.41	0.50	21.31	0.50	-	-	584.20	996.44	~0.00	0.00

Supplementary Table S5. Summary statistics of simple regression analysis (i.e., not fully-combined or derived models) comparing biological data against (ln-transformed) genome size. Most previous studies addressing patterns of genome size diversity in other organisms lack a rigorous analysis accounting for the phylogenetic non-independence of species data[50]. Here we include results from both phylogenetically corrected (PGLS, Phylogenetic Generalized Least Squares, where appropriate) and conventional (OLS, Ordinary Least Squares) regression analyses. This highlights how non-phylogenetically corrected hypothesis testing can artificially increase the likelihood of obtaining a significant result (bold indicates statistically significant results: *$p \leq 0.05$; **$p \leq 0.01$; ***$p \leq 0.001$). For PGLS analysis, each test statistic was calculated using the MCC tree as the main phylogenetic hypothesis, and is followed (in parenthesis) by the median value calculated over the 100 alternative trees. For the *p*-value, the number in parenthesis refers to the percentage of trees with a *p*-value falling on the same side of the significance threshold ($\alpha = 0.05$) as inferred for the MCC tree. Regarding categorical predictors, parameter effects were only summarized using type II sum of squares for simplicity (*F*-test; with details on post hoc pairwise comparisons in Supplementary Tables S7–9). Note that in models containing more than one predictor, interaction terms rendered non-significant results and thus, were excluded from the final analysis (abbreviations, Chondrich.: Chondrichthyes; C_a: Cell area; N_a: Nucleus area; L_{PCA1} (or *L*): Total body length (PCA1 scores); W_{max}: Maximum body weight; *PBS*: Precaudal body shape; *CFAR*: Caudal fin aspect ratio; *SMR*: Standard metabolic rate; *TS*: Tail shape; *k*: Growth completion rate; T_{PCA1}: Age (PCA1 scores); r_{max}: Maximum intrinsic rate of population increase; L_s: Litter size; Reprod. Mode (or *RM*): Reproduction mode; Temperature: Preferred water temperature; Depth (or Dep): Average depth; Clim: Climate; Oc: Occurrence; Sal: Salinity; H_e: Expected heterozygosity; Sp: Species; Gen: Genra).

	PGLS						OLS				
**Model predictors(s)**	***df***	**λ**	**Slope (*β*) ± SE**	***t*-value**	***F*-value**	***p*-value**	***df***	**Slope (*β*) ± SE**	***t*-value**	***F*-value**	***p*-value**
(S1) Lineage (all Chondrich.)	-	-	-	-	-	-	**139**	-	-	**19.040**	**4.92e-08*** **
(S2) Superorder	-	-	-	-	-	-	**68**	-	-	**74.512**	**1.56e-12*** **
(S3) Order	-	-	-	-	-	-	**64**	-	-	**22.573**	**4.26e-14*** **
(S4) Chromosome nº (n)	31	1 (1)	-0.006 ± 0.505 (-0.006)	-0.537 (-0559)	0.289 (0.312)	0.5949 (100%)	31	-0.010 ± 0.011	-0.954	0.909	0.3477
(S5) Fundamental nº (FN)	31	1 (1)	0.0007 ± 0.006 (-0.0001)	0.108 (-0.023)	0.012 (0.014)	0.9145 (100%)	31	-0.007 ± 0.009	-0.863	0.745	0.3946
(S6) Chromosome composition	30	1 (1)	0.038 ± 0.069 (0.035)	0.541 (0.499)	0.293 (0.249)	0.5924 (100%)	28	-0.129 ± 0.075	1.727	2.982	0.0952
(S7) ln-C_a	**31**	**1 (0.96)**	**0.521 ± 0.141 (0.566)**	**3.700 (3.952)**	**13.692 (15.618)**	**8.34e-4*** (100%)**	**31**	**1.032 ± 0.095**	**11.872**	**118.200**	**4.19e-12*** **
(S8) ln-N_a	**22**	**~0 (~0)**	**0.779 ± 0.080 (0.788)**	**9.697 (9.675)**	**94.033 (93.609)**	**2.11e-09*** (100%)**	**22**	**0.779 ± 0.080**	**9.697**	**94.034**	**2.11e-09*** **
(S9) L_{PCA1} + $(L_{PCA1})^2$	67	0.86 (0.84)	*L*: -0.038 ± 0.032 (-0.047)<br>L^2: 0.010 ± 0.009 (0.012)	*L*: -1.183 (-1.410)<br>L^2: 1.125 (1.312)	*L*: 1.400 (1.989)<br>L^2: 1.266 (1.722)	*L*: 0.2510 (100%)<br>L^2: 0.2646 (100%)	**67**	***L*: -0.191 ± 0.042**<br>**L^2: 0.047 ± 0.013**	***L*: -4.580**<br>**L^2: 3.721**	***L*: 20.977**<br>**L^2: 13.849**	***L*: 2.07e-05*** **<br>**L^2: 0.0004*** **
(S10) ln-W_{max}	35	0.88 (0.86)	-0.014 ± 0.021 (-0.019)	-0.667 (-0.809)	0.445 (0.654)	0.5090 (100%)	34	0.005 ± 0027	0.196	0.038	0.8459
(S11) *PBS*	**60**	**0.74 (0.73)**	-	-	**3.086 (3.086)**	**0.0223* (98%)**	**59**	-	-	**41.813**	**2.20e-16*** **
(S12) *CFAR**§	59	0.83 (0.89)	-0.050 ± 0.048 (-0.066)	-1.054 (-1.439)	1.111 (2.070)	0.2962 (59%)	59	-0.090 ± 0.078	-1.177	1.386	0.2438
(S13) ln-*SMR*§	**14**	**0 (0)**	**-0.685 ± 0.103 (-0.685)**	**-6.356 (-6.656)**	**44.300 (44.300)**	**1.09e-05*** (100%)**	**14**	**-0.685 ± 0.103**	**-6.356**	**44.300**	**1.09e-05*** **
(S14) L_{PCA1} + *TS*	**62**	**0.77 (0.72)**	*L*: -0.002 ± 0.023	*L*: -0.083 (-0.103)	*L*: 0.007 (0.1010)	*L*: 0.9344 (100%)	**61**	*L*: 0.007 ± 0.022	*L*: 0.312	*L*: 0.098	*L*: 0.7559

	PGLS						OLS				
**Model predictors(s)**	***df***	**λ**	**Slope (*β*) ± SE**	***t*-value**	***F*-value**	***p*-value**	***df***	**Slope (*β*) ± SE**	***t*-value**	***F*-value**	***p*-value**
(= Cruising Speed)			(-0.002)<br>***TS*: -**	***TS*: -**	***TS*: 3.580 (3.869)**	***TS*: 0.0187* (100%)**		***TS*: -**	***TS*: -**	***TS*: 50.031**	***TS*: 2.00e-16*** **
(S15) k*[§]	54	0.77 (0.80)	-0.144 ± 0.551 (-0.094)	-0.261 (-0.173)	0.068 (0.030)	0.7950 (100%)	**54**	**-2.403 ± 0.796**	**-3.020**	**9.120**	**0.0039** **
(S16) T_{PCA1}*[§]	53	0.84 (0.86)	-0.087 ± 0.073 (-0.083)	-1.203 (-1.158)	1.447 (1.342)	0.2344 (100%)	**53**	**0.246 ± 0.100**	**2.438**	**5.946**	**0.0181** **
(S17) r_{max}[§]	30	0.88 (0.89)	0.244 ± 0.303 (0.319)	0.804 (1.237)	0.647 (1.124)	0.4275 (100%)	30	-0.610 ± 0.442	-1.378	1.898	0.1785
(S18) ln-L_s + Reprod. Mode[§§]	64	0.86 (0.84)	L_s: 0.006 ± 0.037 (0.003)<br>*RM*: -	L_s : 0.163 (0.083)<br>*RM*: -	L_s: 0.027 (0.009)<br>*RM*: 2.111 (2.241)	L_s: 0.8710 (100%)<br>*RM*: 0.1295 (100%)	**62**	L_s: -0.048 ± 0.060<br>***RM*: -**	L_s: -0.807<br>***RM*: -**	L_s: 0.651<br>***RM*: 24.944**	L_s: 0.4230<br>***RM*: 1.13e-08*** **
(S19) Temperature	68	0.89 (0.88)	0.002 ± 0.006 (0.001)	0.323 (0.222)	0.104 (0.072)	0.7479 (100%)	**68**	**-0.034 ± 0.006**	**-6.393**	**40.874**	**1.73e-08*** **
(S20) ln-Depth	69	0.89 (0.87)	-0.009 ± 0.030 (-0.006)	-0.309 (-0.198)	0.095 (0.060)	0.7586 (100%)	**66**	**0.190 ± 0.030**	**6.387**	**40.789**	**1.97e-08*** **
(S21) ln-Depth range	69	0.89 (0.87)	-0.006 ± 0.030 (-0.010)	-0.195 (-0.324)	0.038 (0.105)	0.8463 (100%)	**68**	**0.172 ± 0.042**	**4.101**	**16.815**	**0.0001*** **
(S22) ln-Depth + Climate[†]	65	0.88 (0.85)	Dep: -0.004 ± 0.031 (0.001)<br>Clim: -	Dep: -0.131 (0.046)<br>Clim: -	Dep: 0.017 (0.018)<br>Clim: 0.192 (0.269)	Dep: 0.8959 (98%)<br>Clim: 0.9417 (98%)	**64**	**Dep: 0.114 ± 0.031**<br>**Clim: -**	**Dep: 3.652**<br>**Clim: -**	**Dep: 13.336**<br>**Clim: 6.476**	**Dep: 0.0005*** **<br>**Clim: 0.0002*** **
(S23) ln-Depth + Occurrence[†]	67	0.89 (0.87)	Dep: -0.007 ± 0.032 (0.004)<br>Oc: -	Dep: -0.229 (0.123)<br>Oc: -	Dep: 0.053 (0.040)<br>Oc: 0.057 (0.076)	Dep: 0.8193 (100%)<br>Oc: 0.9446 (100%)	**67**	**Dep: 0.219 ± 0.036**<br>**Oc: -**	**Dep: 6.170**<br>**Oc: -**	**Dep: 38.068**<br>**Oc: 6.710**	**Dep: 4.48e-08*** **<br>**Oc: 0.0022** **
(S24) Habitat	67	0.88 (0.87)	-	-	0.652 (0.775)	0.5846 (100%)	**65**	**-**	**-**	**18.238**	**1.07e-08*** **
(S25) ln-Depth + Salinity[†]	**67**	**0.91 (0.91)**	Dep: -0.019 ± 0.030 (-0.024)<br>**Sal: -**	Dep: -0.668 (-0.816)<br>**Sal: -**	Dep: 0.446 (0.666)<br>**Sal: 5.187 (6.002)**	Dep: 0.5066 (96%)<br>**Sal: 0.0081** (100%)**	**67**	**Dep: 0.117 ± 0.035**<br>**Sal: -**	**Dep: 3.339**<br>**Sal: -**	**Dep: 11.150**<br>**Sal: 5.280**	**Dep: 0.0014** **<br>**Sal: 0.0074** **
(S26) ln-(Nº Sp per Family) + 1 | Family[¶]	69	0.88 (0.87)	0.003 ± 0.038 (0.001)	0.069 (0.037)	0.005 (0.003)	0.9453 (100%)	47<br>(21)	-0.019 ± 0.040<br>(0.077 ± 0.061)	-0.486<br>(1.280)	0.236<br>(1.640)	0.6294<br>(0.2144)
(S27) ln-(Nº Gen per Family) + 1 | Family[¶]	69	0.88 (0.87)	-0.050 ± 0.052 (-0.059)	-0.960 (-1.111)	0.922 (1.233)	0.3402 (100%)	47<br>(21)	-0.098 ± 0.068<br>(-0.033 ± 0.108)	-1.436<br>(-0.302)	2.062<br>(0.091)	0.1576<br>(0.7660)
(S28) ln-(Nº Sp per Order) + 1 | Order[¶]	69	0.88 (0.87)	-0.006 ± 0.067 (-0.005)	-0.093 (-0.067)	0.009 (0.005)	0.9265 (100%)	5<br>(5)	-0.038 ± 0.137<br>(-0.032 ± 0.142)	-0.275<br>(-0.222)	0.076<br>(0.049)	0.7940<br>(0.8329)
(S29) ln-(Nº Gen per Order) + 1 | Order[¶]	69	0.87 (0.86)	-0.092 ± 0.069 (-0.093)	-1.334 (-1.343)	1.780 (1.806)	0.1866 (100%)	5<br>(5)	-0.164 ± 0.108<br>(-0.170 ± 0.108)	-1.516<br>(-1.576)	2.298<br>(2.484)	0.1900<br>(0.1758)
(S30) ln-(Nº Fam per Order) + 1 | Order[¶]	69	0.87 (0.85)	-0.175 ± 0.115 (-0.177)	-1.521 (-1.536)	2.313 (2.359)	0.1329 (100%)	5<br>(5)	-0.262 ± 0.159<br>(-0.270 ± 0.158)	-1.647<br>(-1.706)	2.714<br>(2.912)	0.1604<br>(0.1487)
(S31) H_e[§§]	18	1 (1)	-0.643 ± 0.413 (-0.635)	-1.556 (-1.526)	2.422 (2.330)	0.1371 (86%)	18	-0.493 ± 0.698	-0.707	0.500	0.4887

* Additional results for when poor-quality estimates were omitted (see Supplementary Methods above) are shown in the main text.

§ Body-size correction using regression residuals (i.e., partial correlation analysis). The same conclusion was reached when controlling for body-size via multiple regression, but the first approach was preferred for visual purposes.

§§ Additional body-size corrected results (model S18) and body-size and age corrected results (model S31) via multiple regression are shown in the main text.

† Depth correction performed by including ln-tranformed average depth (“ln-Depth”) as a covariate (i.e., ANCOVA).

¶ Random effect (e.g., “+ 1 | *Taxon*”) incorporated into the OLS regression model only (i.e., mixed-effects linear regression, with parameter estimates reported for the fixed effect only). Results for both cases (OLS and PGLS) were almost identical to the ones following the hierarchical averaging approach of Olmo (2006)[40] (shown in parenthesis under OLS results; see Supplementary Methods above for details).

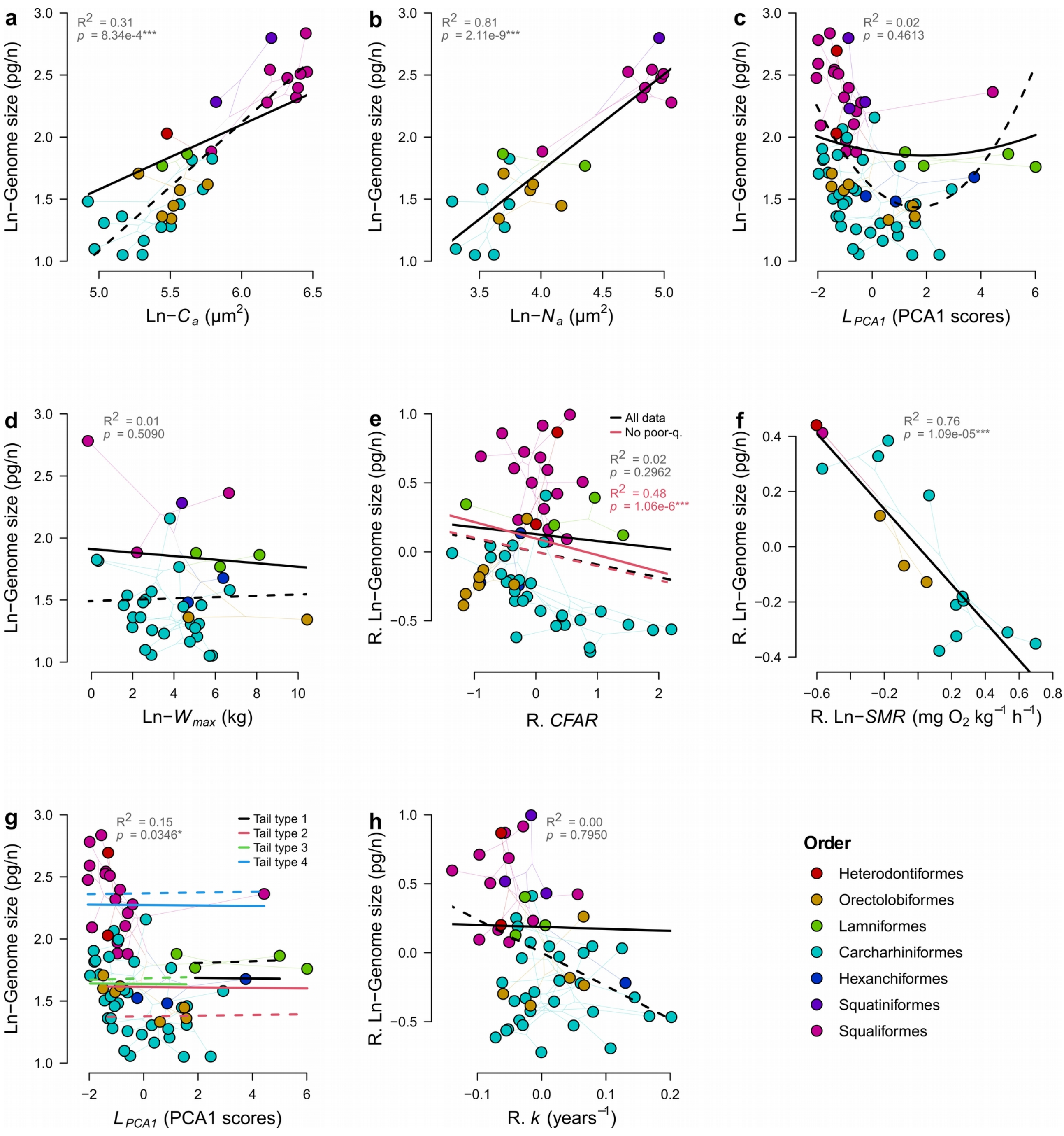

Supplementary Figure S6. Relationships between genome size and cytological, morphological, physiological, and developmental quantitative parameters in sharks. Species mean values for ln-transformed genome size plotted (phylomorphospace) as a function of **a**, Cell size (C_a); **b**, Nucleus size (N_a); **c**, Total body length (PCA1 scores, L_{PCA1}); **d**, Maximum body weight (W_{max}); **e**, Caudal fin aspect ratio (*CFAR*); **f**, Standard metabolic rate (*SMR*); **g**, Cruising speed: "L_{PCA1} + Tail shape (*TS*)"; and **h**, Growth completion rate (*k*). Lines connecting dots (species values) indicate phylogenetic relationships and are colour-coded after taxonomic order (bottom-right inset). Solid black lines represent PGLS regression lines, while dashed black lines represent OLS regression lines (note that for **b** and **f** both lines converge, since $\lambda = 0$ in PGLS regressions). These visualize the results of simple regression analysis (i.e., not fully-combined or derived models; Supplementary Table S5). To achieve linear relationships in *response vs. predictor* comparisons, ln-transformations were applied to C_a, N_a, W_{max}, and *SMR;* while for L_{PCA1} a quadratic term was preferred for a better model-fit (since ln-transformation was not possible due to the existence of negative values in the PCA scores). For *CFAR*, *SMR*, and *k*, body-size corrections were applied by comparing, as shown

here, the regression residuals ("R.") of each parameter against L_{PCA1} (i.e., partial correlation analysis).

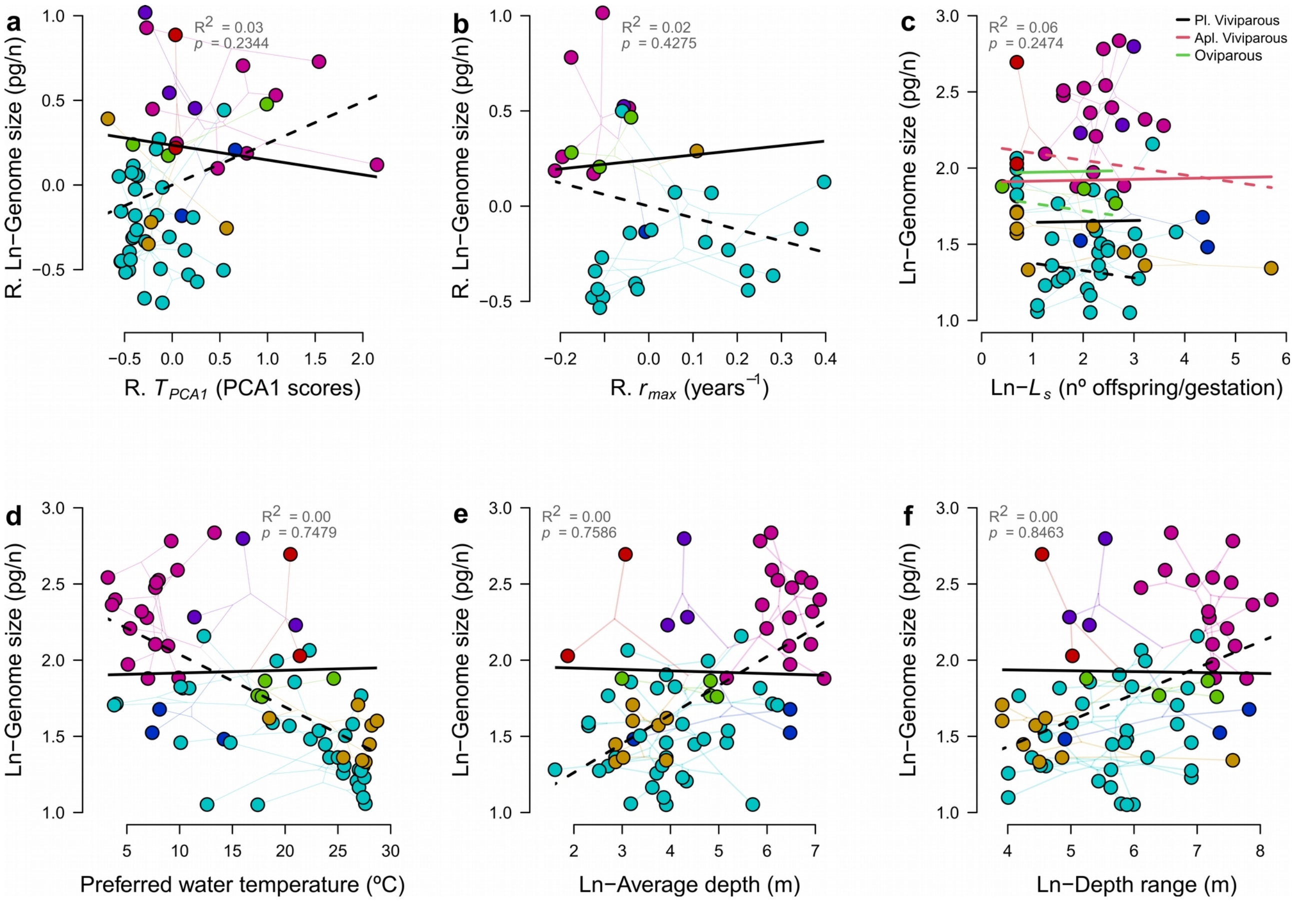

Supplementary Figure S7. Relationships between genome size and developmental, demographic, reproductive, and ecological quantitative parameters in sharks. Species mean values for ln-transformed genome size plotted (phylomorphospace) as a function of **a**, Age (PCA1 scores, T_{PCA1}); **b**, Maximum intrinsic rate of population increase (r_{max}); **c**, Reproduction: "Litter size (L_s) + Reproduction mode"; **d**, Preferred water temperature; **e**, Average depth; and **f**, Depth range. Lines connecting dots (species values) indicate phylogenetic relationships and are colour-coded after taxonomic order as in Supplementary Fig. S6. Solid black lines represent PGLS regression lines, while dashed black lines represent OLS regression lines. These visualize the results of simple regression analysis (Supplementary Table S5). To achieve linear relationships in *response vs. predictor* comparisons, ln-transformations were applied to L_s, Average depth, and Depth range. For T_{PCA1} and r_{max}, body-size corrections were applied by comparing, as shown here, the regression residuals ("R.") of each parameter against L_{PCA1} (i.e., partial correlation analysis).

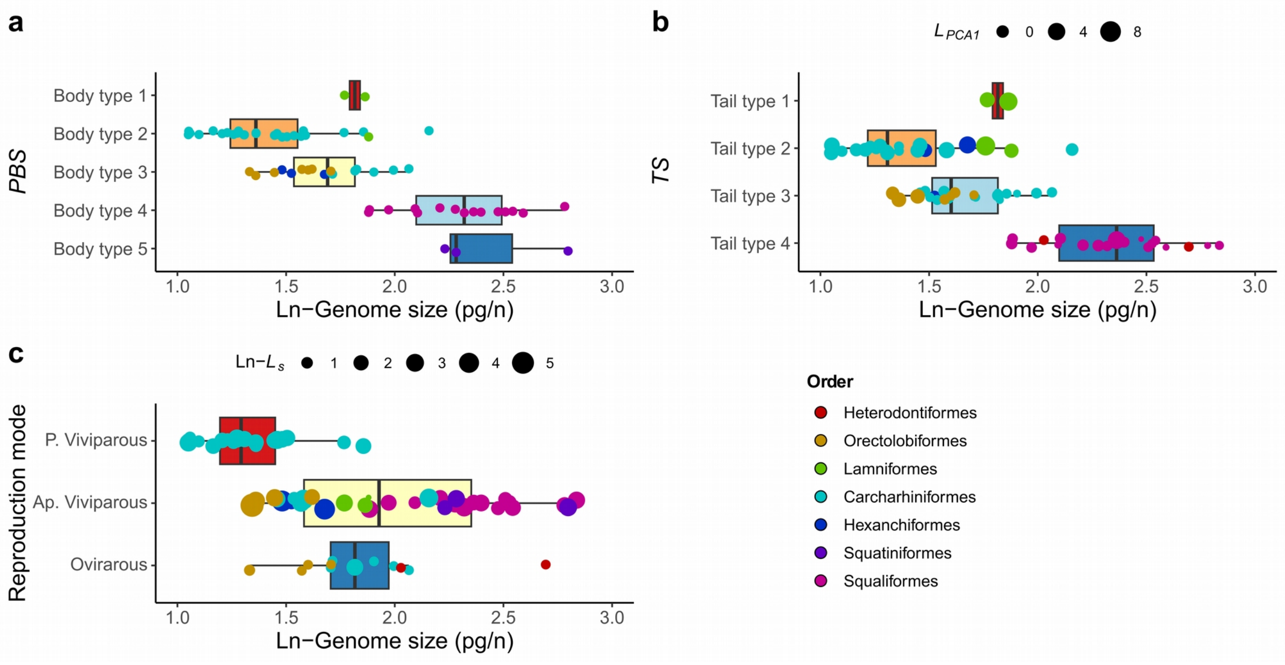

Supplementary Figure S8. Genome size diversity across morphological, physiological, and reproductive qualitative parameters in sharks. Distribution of ln-transformed genome size plotted (boxplot) as a function of **a**, Precaudal body shape (*PBS*); **b**, Tail shape (*TS*); and **c**, Reproduction mode. Individual jitter points depict average values per species and are colour-coded after taxonomic order (bottom-right inset). Jitter dot-size proportional to body size (L_{PCA1}) for **b**, and to ln-transformed litter size (Ln-L_s) for **c**, in concomitance with the simple regression models analysed (Supplementary Table S5).

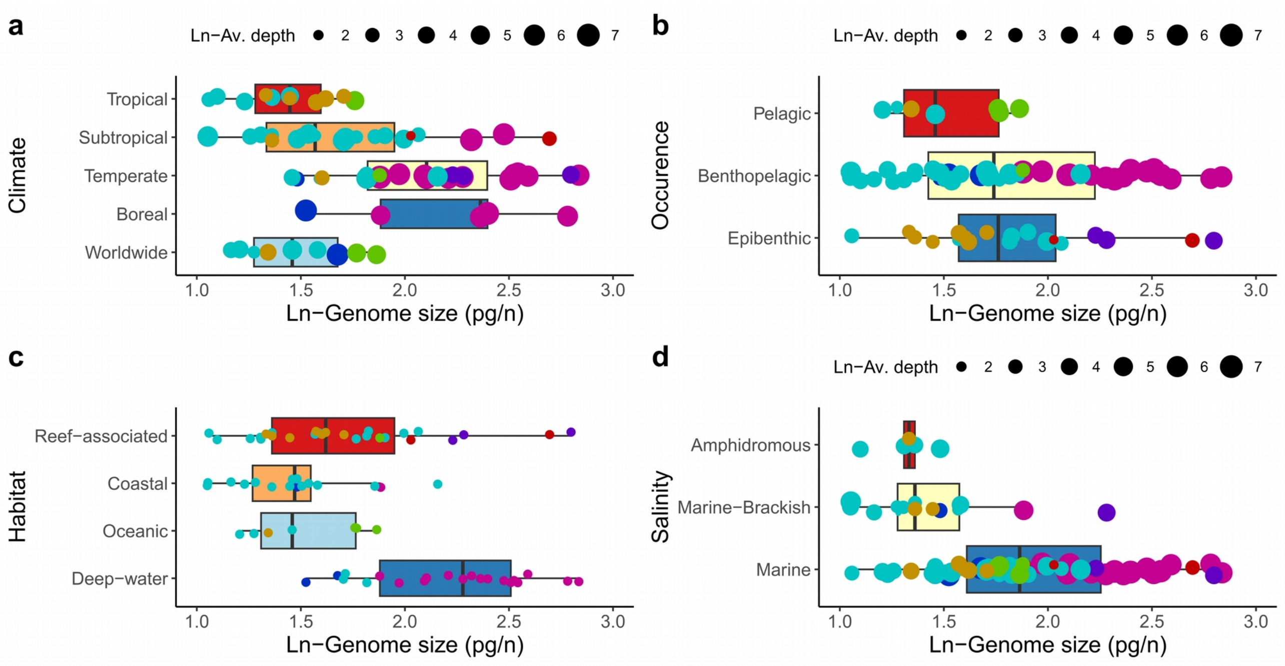

Supplementary Figure S9. Genome size diversity across ecological qualitative parameters in sharks. Distribution of ln-transformed genome size plotted (boxplot) as a function of **a**, Climate; **b**, Occurrence across the water column; **c**, Habitat; and **d**, Salinity. Individual jitter points depict average values per species and are colour-coded after taxonomic order as in Supplementary Fig. S8.

Jitter dot-size proportional to ln-transformed average depth (Ln-Av. depth) for **a**, **b**, and **d**, in concomitance with the simple regression models analysed (Supplementary Table S5).

Supplementary Table S6. Model averaging results for the best minimum adequate multivariate PGLS models from Table 3. This step is performed over the set of best models obtained (based on AICc minimization) from the same fully-combined model (models 1 to 4 in Table 3). Parameter estimates are averaged only over the models where the given parameter appears (i.e., conditional average). Model numbers and abbreviations as in Table 3. Details regarding post hoc pairwise comparisons for categorical predictors are given in Supplementary Tables S7-S10.

**Best models**	**Predictors**	**Slope (*β*)**	**SE**	***Z*-value**	***Pr* (>|*z*|)**
1.1) 1 + L_{PCA1} + *PBS* + *RM* + ln-L_s + Sal<br>1.2) 1 + L_{PCA1} + *PBS* + *RM* + Sal<br>1.3) 1 + L_{PCA1} + *PBS* + ln-L_s + Sal<br>1.4) 1 + L_{PCA1} + *PBS* + Sal<br>1.5) 1 + L_{PCA1} + *PBS* + *RM* + ln-L_s + ln-Depth + Sal<br>1.6) 1 + L_{PCA1} + ln-L_s + Sal<br>1.7) 1 + L_{PCA1} + Sal	**Intercept**	**1.338**	**0.283**	**4.722**	**2.30e-06*** **
	L_{PCA1}	0.002	0.024	0.099	0.9209
	*PBS*_Body type 2	0.006	0.213	0.027	0.9784
	*PBS*_Body type 3	-0.044	0.245	0.181	0.8566
	***PBS*_Body type 4**	**0.555**	**0.267**	**2.077**	**0.038***
	***PBS*_Body type 5**	**0.797**	**0.307**	**2.592**	**0.0095****
	***RM*_Aplacental viviparous**	**0.256**	**0.105**	**2.430**	**0.0151***
	***RM*_Oviparous**	**0.410**	**0.177**	**2.313**	**0.0207***
	ln-L_s	0.071	0.041	1.753	0.0797
	ln-Depth	-0.040	0.031	1.311	0.1900
	Sal_Marine-Brackish	-0.036	0.103	0.347	0.7287
	**Sal_Marine**	**0.216**	**0.095**	**2.285**	**0.0223***
Model averaging for the best models derived form model 2 as for models 1.1 to 1.7 (above).					
3.1) 1 + L_{PCA1} + *TS* + Sal<br>3.2) 1 + L_{PCA1} + *TS* + *RM* + Sal<br>3.3) 1 + L_{PCA1} + *TS* + ln-L_s + Sal<br>3.4) 1 + L_{PCA1} + *TS* + *RM* + ln-L_s + Sal<br>3.5) 1 + L_{PCA1} + Sal	**Intercept**	**1.377**	**0.297**	**4.631**	**3.60e-06*** **
	L_{PCA1}	0.002	0.026	0.078	0.9382
	*TS*_Tail type 2	0.001	0.229	0.006	0.9954
	*TS*_Tail type 3	-0.030	0.249	0.119	0.9052
	***TS*_Tail type 4**	**0.584**	**0.286**	**2.039**	**0.0414***
	***RM*_Aplacental viviparous**	**0.251**	**0.118**	**2.126**	**0.0335***
	***RM*_Oviparous**	**0.310**	**0.149**	**2.088**	**0.0368***
	ln-L_s	0.050	0.042	1.181	0.2378
	Sal_Marine-Brackish	-0.010	0.101	0.096	0.9236
	**Sal_Marine**	**0.223**	**0.100**	**2.241**	**0.0250***
Model averaging for the best models derived form model 4 as for models 3.1 to 3.5 (above).					

Significant results in bold (*$p \leq 0.05$; **$p \leq 0.01$; ***$p \leq 0.001$).

Supplementary Table S7. Post hoc pairwise comparisons between the five shark precaudal body shape (*PBS*) types and their effect on genome size. Parameter estimates are based on model S11 (from Supplementary Table S5). Post hoc analysis performed on the conditional average of models 1.1 to 1.5 (from Supplementary Table S6, where *PBS* was included among the best minimum adequate multivariate models) rendered very similar parameter estimates and thus, only the *p*-values are included in parenthesis.

	**Pairwise comparison**			
**Reference level (Intercept ↓)**	**Body type 2**	**Body type 3**	**Body type 4**	**Body type 5**
**Body type 1**	β = -0.134 ± 0.233<br>t = -0.577, p = 0.6293<br>(0.9784)	β = 0.0004 ± 0.241<br>t = 0.002, p = 0.9985<br>(0.9520)	B = 0.559 ± 0.300<br>t = 1.862, p = 0.1124<br>(0.0630)	β = 0.778 ± 0.346<br>t = 2.249, p = 0.0565<br>(**0.0191***)
**Body type 2**		β = 0.135 ± 0.157<br>t = 0.859, p = 0.5043<br>(0.9520)	**β = 0.693 ± 0.243**<br>**t = 2.853, p = 0.0207***<br>**(0.0191*)**	**β = 0.913 ± 0.298**<br>**t = 3.062, p = 0.0207***<br>**(0.0068**)**
**Body type 3**			**β = 0.558 ± 0.213**<br>**t = 2.620, p = 0.0278***<br>**(0.0068**)**	**β = 0.778 ± 0.274**<br>**t = 2.836, p = 0.0207***<br>**(0.0027**)**
**Body type 4**				β = 0.219 ± 0.261<br>t = 0.842, p = 0.5043<br>(0.3822)

Significant results in bold (*$p \leq 0.05$; **$p \leq 0.01$; ***$p \leq 0.001$).
P-values adjusted for multiple comparisons via False Discovery Rates (FDR).

Supplementary Table S8. Post hoc pairwise comparisons between the four shark tail shape (*TS*) types and their effect on genome size. Parameter estimates are based on model S14 (from Supplementary Table S5). Post hoc analysis performed on the conditional average of models 3.1 to 3.4 (from Supplementary Table S6, where *TS* was included among the best minimum adequate multivariate models) rendered very similar parameter estimates and thus, only the *p*-values are included in parenthesis.

	**Pairwise comparison**		
**Reference level (Intercept ↓)**	**Tail type 2**	**Tail type 3**	**Tail type 4**
**Tail type 1**	β = -0.076 ± 0.231, t = -0.328,<br>p = 0.8585 (0.9954)	β = -0.053 ± 0.253, t = -0.208,<br>p = 0.8585 (0.9954)	β = 0.583 ± 0.303, t = 1.926,<br>p = 0.1175 (0.0828)
**Tail type 2**		β = 0.023 ± 0.130, t = 0.179,<br>p = 0.8585 (0.9954)	**β = 0.659 ± 0.217, t = 3.036,**<br>**p = 0.0105* (0.0092**)**
**Tail type 3**			**β = 0.636 ± 0.200, t = 3.175,**<br>**p = 0.0105* (0.0031**)**

Significant results in bold (*$p \leq 0.05$; **$p \leq 0.01$; ***$p \leq 0.001$).
P-values adjusted for multiple comparisons via False Discovery Rates (FDR).

Supplementary Table S9. Post hoc pairwise comparisons between the three water salinity environments inhabited by sharks and their effect on genome size. Parameter estimates are based on model S25 (from Supplementary Table S5). Post hoc analysis performed on the conditional average of models 1.1 to 1.7 and 3.1 to 3.5 (from Supplementary Table S6, where salinity was included among the best minimum adequate multivariate models) rendered similar parameter estimates and thus, only the *p*-values are included in parenthesis (in the same order as described above).

	**Pairwise comparison**	
**Reference level (Intercept ↓)**	**Marine-Brackish**	**Marine**
**Amphidromous**	$\beta = 0.009 \pm 0.092$<br>$t = 0.098$, $p = 0.9225$ (0.7287, 0.9236)	**$\beta = 0.210 \pm 0.094$<br>$t = 2.238$, $p = 0.0429$* (0.0335*, 0.0375*)**
**Marine-Brackish**		**$\beta = 0.201 \pm 0.067$<br>$t = 3.005$, $p = 0.0112$* (0.0022**, 0.0066**)**

Significant results in bold (*$p \leq 0.05$; **$p \leq 0.01$; ***$p \leq 0.001$).
P-values adjusted for multiple comparisons via False Discovery Rates (FDR).

Supplementary Table S10. Post hoc pairwise comparisons between the three reproduction modes adopted by sharks and their effect on genome size. Parameter estimates are based on the conditional average of models 1.1, 1.2, and 1.5 (from Supplementary Table S6, where reproduction mode was included among the best minimum adequate multivariate models).

	**Pairwise comparison**	
**Reference level (Intercept ↓)**	**Aplacental viviparous**	**Oviparous**
**Placental viviparous**	**$\beta = 0.256 \pm 0.105$, $p = 0.0311$***	**$\beta = 0.410 \pm 0.177$, $p = 0.0311$***
**Aplacental viviparous**		$\beta = 0.153 \pm 0.155$, $p = 0.3212$

Significant results in bold (*$p \leq 0.05$; **$p \leq 0.01$; ***$p \leq 0.001$).
P-values adjusted for multiple comparisons via False Discovery Rates (FDR).

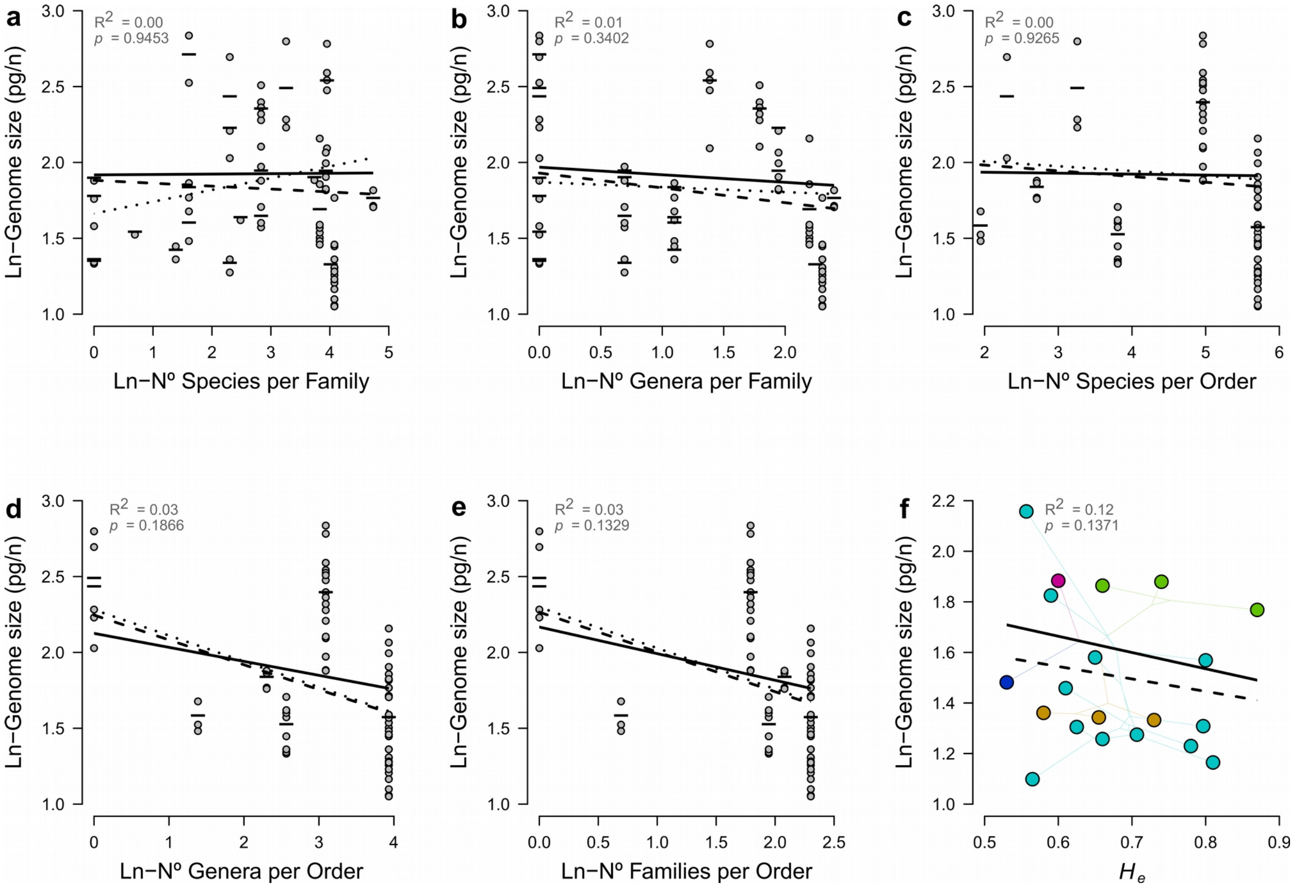

Supplementary Figure S10. Relationships between genome size and taxonomic and genetic diversity parameters in sharks. Species mean values for ln-transformed genome size plotted as a function of ln-transformed **a**, Number of species per family; **b**, Number of genera per family; **c**, Number of species per order; **d**, Number of genera per order; **e**, Number of families per order; and **f**, expected heterozygosity (H_e, not ln-transformed). For **a**–**e**, solid black lines represent PGLS regression lines, long dashed black lines represent mixed-effects linear regression lines (restricted to the fixed term; Supplementary Table S5), while short dashed black lines represent OLS regression lines on averaged values (hyphens) at the respective taxonomic scale (i.e., family for **a** and **b**, and order for **c**–**e**), following Olmo (2006)[40]. For **f**, lines connecting dots (species values) indicate phylogenetic relationships and are colour-coded after taxonomic order (as elsewhere), solid black lines represent PGLS regression lines, while dashed black lines represent OLS regression lines.

Supplementary Table S11. Comparison of genome size estimates obtained through sequencing-based and non-sequencing-based techniques. Sequencing-based genome size (GS) estimates, expressed in Gb, correspond to whole-genome assembly lengths (WG) and k-mers (where available). Genome size estimates obtained with non-sequencing-based techniques are taken from Supplementary Table S1 averages, expressed also in Gb (for which original estimates and references can be found in Supplementary Data S1; measurement technique abbreviations: FIA: Feulgen Image Analysis Densitometry; FCM: Flow Cytometry; FD: Feulgen Densitometry; BFA: Bulk Fluorometric Assay).

Species	Sequencing-based GS estimate	Non-sequencing-based GS estimate
Hemiscyllium ocellatum	3.98 Gb (male; WG) / 4.15 Gb (female; WG)[51]	5.39 Gb (= 5.51 pg; FIA)
Chiloscyllium plagiosum	3.85 Gb (WG)[52]<br>4.99 Gb (domestic; k-mer) / ~3.99 Gb (wild; k-mer)[53]	4.85 Gb (= 4.96 pg; FCM)
Chiloscyllium punctatum	3.38 Gb (WG)[54]	4.71 Gb (= 4.82 pg; FCM, FIA, qPCR)
Rhincodon typus	3.44 Gb (k-mer)[55]<br>2.66 Gb (WG)[54]<br>3.20 Gb (WG)[56]<br>3.13 Gb (k-mer)[56]<br>2.96 Gb (WG)[57]<br>~2.79 Gb (k-mer)[57]<br>2.88 Gb (annotated WG)[58]	3.75 Gb (= 3.83 pg; FCM)
Stegostoma tigrinum	1.70 Gb (k-mer)[59]<br>2.77 Gb (annotated WG)[58]	3.71 Gb (=3.79 pg; FCM)
Carcharodon carcharias	4.08 Gb (WG)[60]<br>4.63 Gb (k-mer)[60]	6.31 Gb (=6.45 pg; FCM)
Isurus oxyrinchus	4.98 Gb (WG)[61]	5.73 Gb (= 5.86 pg; FD, FIA)
Scyliorhinus torazame	4.47 Gb (WG)[54]	6.57 Gb (= 6.72 pg; FCM, FD)
Negaprion brevirostris	2.29–2.58 Gb (k-mer)[62]	3.62 Gb (= 3.70 pg; BFA)
Squalus acanthias	3.70 Gb (WG)[63]	6.44 Gb (= 6.58 pg; FCM, FD, FIA, BFA)

References for Supplementary Information I

1. Gregory, T. R. Cell Size Database. *World Wide Web electronic publication* www.genomesize.com (2005).
2. Emery, S. H. Hematological comparisons of endothermic vs ectothermic elasmobranch fishes. *Copeia* **1986**, 700 (1986).
3. Neale, N. L., Honn, K. V. & Chavin, W. Hematological responses to thermal acclimation in a cold water squaliform (*Heterodontus francisci* Girard). *J. Comp. Physiol. B.* **115**, 215–222 (1977).
4. Dove, A., Arnold, J. & Clauss, T. Blood cells and serum chemistry in the world's largest fish: the whale shark *Rhincodon typus*. *Aquat. Biol.* **9**, 177–183 (2010).
5. Ebert, D. A., Fowler, S. & Compagno, L. *Sharks of the World: A Fully Illustrated Guide* (Wild Nature Press, 2013).
6. Froese, R. & Pauly, D. Fishbase. Version 02/2024. *World Wide Web electronic publication* www.fishbase.org (2024).
7. Pollerspöck, J. & Straube, N. Bibliography database of living/fossil sharks, rays and chimaeras (Chondrichthyes: Elasmobranchii, Holocephali) - List of Valid Extant Species;

List of Described Extant Species; Statistic. Version 08/2023. *World Wide Web electronic publication* www.shark-references.com (2023).

8. Sternes, P. C. & Shimada, K. Body forms in sharks (Chondrichthyes: Elasmobranchii) and their functional, ecological, and evolutionary implications. *Zoology* **140**, 125799 (2020).

9. Thomson, K. S. & Simanek, D. E. Body form and locomotion in sharks. *American Zoologist* **17**, 343–354 (1977).

10. Sambilay, V. C. Interrelationships between swimming speed, caudal fin aspect ratio and body length of fishes. *Fishbyte* **8**, 16–20 (1990).

11. Iliou, A. S. *et al.* Tail shape and the swimming speed of sharks. *R. Soc. Open Sci.* **10**, 231127 (2023).

12. Scacco, U., La Mesa, G. & Vacchi, M. Body morphometrics, swimming diversity and niche in demersal sharks: a comparative case study from the Mediterranean Sea. *Sci. Mar.* **74**, 37–53 (2010).

13. Carrier, J. C., Musick, J. A. & Heithaus, M. R. *Biology of Sharks and their Relatives. Second edition* (CRC Press, 2012).

14. Routley, M. H., Nilsson, G. E. & Renshaw, G. M. C. Exposure to hypoxia primes the respiratory and metabolic responses of the epaulette shark to progressive hypoxia. *Comp. Biochem. Physiol. A. Mol. Integr. Physiol.* **131**, 313–321 (2002).

15. Barnett, A., Payne, N. L., Semmens, J. M. & Fitzpatrick, R. Ecotourism increases the field metabolic rate of whitetip reef sharks. *Biol. Conserv.* **199**, 132–136 (2016).

16. Lear, K. O., Gleiss, A. C. & Whitney, N. M. Metabolic rates and the energetic cost of external tag attachment in juvenile blacktip sharks *Carcharhinus limbatus*. *J. Fish Biol.* **93**, 391–395 (2018).

17. Luongo, S. M. & Lowe, C. G. Seasonally acclimated metabolic Q10 of the california horn shark, *Heterodontus francisci*. *J. Exp. Mar. Bio. Ecol.* **503**, 129–135 (2018).

18. White, C. R., Phillips, N. F. & Seymour, R. S. The scaling and temperature dependence of vertebrate metabolism. *Biol. Lett.* **2**, 125–127 (2006).

19. Ryan, L. A., Meeuwig, J. J., Hemmi, J. M., Collin, S. P. & Hart, N. S. It is not just size that matters: shark cruising speeds are species-specific. *Mar. Biol.* **162**, 1307–1318 (2015).

20. De Wysiecki, A. M. & Braccini, J. M. Shark length–length relationships: studying morphology allows the detection of bias in routine fisheries sampling. *Reg. Stud. Mar. Sci.* **16**, 290–293 (2017).

21. von Bertalanffy, L. Untersuchungen Über die Gesetzlichkeit des Wachstums - I. Teil: Allgemeine Grundlagen der Theorie; Mathematische und physiologische Gesetzlichkeiten des Wachstums bei Wassertieren. *Wilhelm Roux. Arch. Entwickl. Mech. Org.* **131**, 613–652 (1934).

22. Smith, S. E., Au, D. W. & Show, C. Intrinsic rebound potentials of 26 species of pacific sharks. *Mar. Freshw. Res.* **49**, 663–678 (1998).

23. Cortés, E. Life history patterns and correlations in sharks. *Rev. Fish. Sci.* **8**, 299–344 (2000).

24. Cailliet, G. M. *et al.* Age determination and validation studies of marine fishes: do deep-dwellers live longer? *Experimental Gerontology* **36**, 739–764 (2001).

25. Frisk, M. G., Miller, T. J. & Fogarty, M. J. Estimation and analysis of biological parameters in elasmobranch fishes: a comparative life history study. *Can. J. Fish. Aquat. Sci.* **58**, 969–981 (2001).

26. Cortés, E. Incorporating uncertainty into demographic modeling: application to shark populations and their conservation. *Conserv. Biol.* **16**, 1048–1062 (2002).

27. Cailliet, G. M. & Goldman, K. J. Age determination and validation in chondrichthyan fishes in *Biology of Sharks and Their Relatives* (eds. Carrier, J. C., Musick, J. A. & Heithaus, M. R.) 399–447 (CRC Press, 2004).

28. Carlson, J. K. & Goldman, K. J. *Special Issue: Age and Growth of Chondrichthyan Fishes: New Methods, Techniques and Analysis* (Springer, 2006).

29. Cope, J. M. Exploring intraspecific life history patterns in sharks. *Fish. Bull.* **104**, 311–320 (2006).

30. García, V. B., Lucifora, L. O. & Myers, R. A. The importance of habitat and life history to extinction risk in sharks, skates, rays and chimaeras. *Proc. Royal Soc. B.* **275**, 83–89 (2008).

31. Camhi, M. D., Pikitch, E. K. & Babcock, E. A. *Sharks of the Open Ocean: Biology, Fisheries and Conservation* (Wiley-Blackwell, 2008).

32. Carrier, J. C., Musick, J. A. & Heithaus, M. R. *Sharks and Their Relatives II: Biodiversity, Adaptive Physiology, and Conservation* (CRC Press, 2010).

33. De Magalhães, J. P. & Costa, J. A database of vertebrate longevity records and their relation to other life-history traits. *J. Evol. Biol.* **22**, 1770–1774 (2009).

34. IUCN 2023. The IUCN Red List of Threatened Species. Version 1/2023. *World Wide Web electronic publication* www.iucnredlist.org (2023).

35. Pardo, S. A., Kindsvater, H. K., Reynolds, J. D. & Dulvy, N. K. Maximum intrinsic rate of population increase in sharks, rays, and chimaeras: the importance of survival to maturity. *Can. J. Fish. Aquat. Sci.* **73**, 1159–1163 (2016).

36. Ohta, T. & Kimura, M. A model of mutation appropriate to estimate the number of electrophoretically detectable alleles in a finite population. *Genet. Res.* **22**, 201–204 (1973).

37. Domingues, R. R., Hilsdorf, A. W. S. & Gadig, O. B. F. The importance of considering genetic diversity in shark and ray conservation policies. *Conservation Genetics* **19**, 501–525 (2018).

38. Fox, J. & Weisberg, S. *An R Companion to Applied Regressions. Third edition* (Sage, 2019).

39. Pinheiro, J. & Bates, D. M. nlme: Linear and Nonlinear Mixed Effects Models. R package version 3.1-164, https://CRAN.R-project.org/package=nlme (2023).

40. Olmo, E. Genome size and evolutionary diversification in vertebrates. *Ital. J. Zool.* **73**, 167–171 (2006).

41. Levan, A., Fredga, K. & Sandberg, A. A. Nomenclature for centromeric position on chromosomes. *Hereditas* **52**, 201–220 (1964).

42. Schwartz, F. J. & Maddock, M. B. Cytogenetics of the elasmobranchs: genome evolution and phylogenetic implications. *Marine and Freshwater Research* **53**, 491–502 (2002).

43. Da Silva Rodrigues-Filho, L. F. *et al.* Evolutionary history and taxonomic reclassification of the critically endangered daggernose shark, a species endemic to the western atlantic. *J. Zool. Syst. Evol. Res.* **2023**, 4798805 (2023).

44. Nishimura, O. *et al.* Squalomix: shark and ray genome analysis consortium and its data sharing platform. *F1000Research* **11**, 1077 (2022).

45. Schwartz, F. J. & Maddock, M. B. Comparisons of karyotypes and cellular dna contents within and between major lines of elasmobranches in 'Indo-Pacific Fish Biology. Proceedings of the 2nd International Conference in Indo–Pacific Fishes', pp. 148–157. Ichthyological Society of Japan, Tokyo (1986).

46. Peterson, B. G. & Carl, P. PerformanceAnalytics: Econometric Tools for Performance and Risk Analysis. R Package Version 2.0.4. https://CRAN.R-project.org/package=PerformanceAnalytics (2020).

47. Xiao, N. ggsci: Scientific Journal and Sci-Fi Themed Color Palettes for 'ggplot2'. R package version 3.0.0, https://CRAN.R-project.org/package=ggsci (2023).

48. Wilke, C. O. ggridges: Ridgeline Plots in 'ggplot2'. R package version 0.5.6, https://CRAN.R-project.org/package=ggridges (2024).

49. Xu, S. *et al.* Use ggbreak to effectively utilize plotting space to deal with large datasets and outliers. *Front. Genet.* **12**, 774846 (2021).

50. Gregory, T. R. Genome size evolution in animals in *The Evolution of the Genome* (ed. Gregory, T. R.) 3–87 (Academic Press, 2005).

51. Sendell-Price, A. T. *et al.* Low mutation rate in epaulette sharks is consistent with a slow rate of evolution in sharks. *Nat. Commun.* **14**, 6628 (2023).

52. Zhang, Y. *et al.* The white-spotted bamboo shark genome reveals chromosome rearrangements and fast-evolving immune genes of cartilaginous fish. *IScience* **23**, 101754 (2020).

53. Zhao, R. *et al.* Genomic comparison and genetic marker identification of the white-spotted bamboo shark *Chiloscyllium plagiosum*. *Front. Mar. Sci.* **9**, 936681 (2022).

54. Hara, Y. *et al.* Shark genomes provide insights into elasmobranch evolution and the origin of vertebrates. *Nat. Ecol. Evol.* **2**, 1761–1771 (2018).

55. Read, T. D. *et al.* Draft sequencing and assembly of the genome of the world's largest fish, the whale shark: *Rhincodon typus* Smith 1828. *BMC Genomics* **18**, 532 (2017).

56. Weber, J. A. *et al.* The whale shark genome reveals how genomic and physiological properties scale with body size. *Proc. Natl. Acad. Sci. U. S. A.* **117**, 20662–20671 (2020).

57. Tan, M. *et al.* The whale shark genome reveals patterns of vertebrate gene family evolution. *Elife* **10**, e65394 (2021).

58. Yamaguchi, K. *et al.* Elasmobranch genome sequencing reveals evolutionary trends of vertebrate karyotype organization. *Genome Res.* **33**, 1527–1540 (2023).

59. Kadota, M. *et al.* Shark and ray genome size estimation: methodological optimization for inclusive and controllable biodiversity genomics. *F1000Research* **12**, 1204 (2023).

60. Marra, N. J. *et al.* White shark genome reveals ancient elasmobranch adaptations associated with wound healing and the maintenance of genome stability. *Proc. Natl. Acad. Sci. U. S. A.* **116**, 4446–4455 (2019).

61. Stanhope, M. J. *et al.* Genomes of endangered great hammerhead and shortfin mako sharks reveal historic population declines and high levels of inbreeding in great hammerhead. *IScience* **26**, 105815 (2022).

62. Baeza, J. A. Insights into the nuclear and mitochondrial genome of the Lemon shark *Negaprion brevirostris* using low-coverage sequencing: genome size, repetitive elements, mitochondrial genome, and phylogenetic placement. *Gene* **894**, 147939 (2023).

63. Wagner, C. I. *et al.* Characteristics of the spiny dogfish (*Squalus acanthias*) nuclear genome. *G3 Genes, Genomes, Genet.* **13**, jkad146 (2023).
